# Genetic loci regulating the concentrations of anthocyanins and proanthocyanidins in the pericarps of purple and red rice

**DOI:** 10.1002/tpg2.20338

**Published:** 2023-05-13

**Authors:** Ming‐Hsuan Chen, Shannon R. M. Pinson, Aaron K. Jackson, Jeremy D. Edwards

**Affiliations:** ^1^ Dale Bumpers National Rice Research Center, United States Department of Agriculture—Agricultural Research Service Stuttgart AR USA

## Abstract

The pigmented flavonoids, anthocyanins and proanthocyanidins, have health promoting properties. Previous work determined that the genes *Pb* and *Rc* turn on and off the biosynthesis of anthocyanins (purple) and proanthocyanidins (red), respectively. Not yet known is how the concentrations of these pigmented flavonoids are regulated in grain pericarps. Quantitative trait locus (QTL) analysis in a population of rice (*Oryza sativa* L.) F5 recombinant inbred lines from white pericarp “IR36ae” x red+purple pericarp “242” revealed three QTLs associated with grain concentrations of anthocyanins (TAC) or proanthocyanidins (PA). Both TAC and PA independently mapped to a 1.5 Mb QTL region on chromosome 3 between RM3400 (at 15.8 Mb) and RM15123 (17.3 Mb), named *qPR3*. Across 2 years, *qPR3* explained 36.3% of variance in TAC and 35.8% in PA variance not attributable to *Pb* or *Rc*. The *qPR3* region encompasses *Kala3*, a MYB transcription factor previously known to regulate purple grain characteristics. Study of *PbPbRcrc* progeny showed that TAC of *RcRc* near isogenic lines (NILs) was 2.1–4.5x that of *rcrc*. Similarly, study of *PbPbRcRc* NILs, which had 70% higher PA than *pbpbRcRc* NILs, revealed a mutual enhancement, not a trade‐off between these compounds that share precursors. This suggests that *Pb* and *Rc* upregulate genes in a shared pathway as they activate TAC and PA synthesis, respectively. This study provides molecular markers for facilitating marker‐assisted selection of *qPR3*, *qPR5*, and *qPR7* to enhance grain concentrations of pigmented flavonoids and documented that stacking *Rc* and *Pb* genes further increases both flavonoid compounds.

## Core Ideas

- Anthocyanins and proanthocyanidins are pigmented flavonoids with desirable health‐promoting properties.
- *Pb* and *Rc* are transcription factors that activate biosynthesis of anthocyanins and proanthocyanidins, respectively.
- Mutual enhancement was discovered, with *Rc* increasing anthocyanin content and *Pb* increasing proanthocyanidins.
- Three QTLs were newly identified as regulating concentrations of anthocyanins and/or proanthocyanidins.
- *Pb*, *Rc*, and these molecularly tagged QTLs can be used to enhance the nutritional quality of rice varieties

**Abbreviations**
ANOVA: Analysis of variance
ANR: anthocyanidin reductase
ANS: Anthocyanidin synthase
bHLH: beta helix‐loop‐helix
BLUP: best linear unbiased predictor
CIM: composite interval mapping
DFR: dihydroflavonol 4‐reductase
DMAC: 4‐dimethylaminocinamaldeyde
EBGs: early biosynthesis genes
InDel: molecular marker tagging an insertion/deletion site
LAR: leucoanthocyanidin reductase; LBGs, late biosynthesis genes
LDOX: leucoanthocyanidin dioxygenase
LOD: Log‐of‐odds
LSD: least significant difference
MBW: a 3‐protein complex formed from a MYB, a beta helix‐loop‐helix (bHLH), and a WD40 protein
MIM: multiple interval mapping
NIL: near isogenic line
PA: total concentration of proanthocyanidins
PAs: proanthocyanidins
PCR: Polymerase chain reaction
QTL: quantitative trait locus
RIL: recombinant inbred line
SMR: single marker regression
SNP: single‐nucleotide polymorphism
SSR: simple sequence repeat molecular marker
TAC: total concentration of all anthocyanins
TF: transcription factor
UGT: UDP‐glucosyl transferase

## 1 INTRODUCTION

With increasing global health challenges in the incidence of chronic diseases (e.g., heart disease, type II diabetes, obesity, and cancers), nutrient dense whole grain cereals, including rice (*Oryza sativa* L.), have the potential to contribute to their prevention (Dipti et al., 2012; Jones & Engleson, 2010). Rice contains health beneficial compounds, such as simple phenolics, flavonoids, and lipophilic antioxidants, specifically tocopherols, tocotrienols, and γ‐oryzanols (Chen et al., 2016, 2006, 2017; Min et al., 2011). These compounds are primarily deposited in the bran layer (pericarp, aleurone, and germ) of the whole grain rice (Dipti et al., 2012). Among these phytonutrients, the pigmented flavonoids anthocyanins and proanthocyanidins (PAs) in purple and red pigmented rices, respectively, have been associated with several health benefits including anti‐inflammatory and anti‐cancer activities, cardiovascular disease prevention, diabetes alleviation, and obesity control, as well as having antioxidant capacity (Boue et al., 2016; Chen et al., 2023; González‐Abuín et al., 2015; He & Giusti, 2010). Development of nutrient dense rice cultivars with higher contents of these health promoting flavonoids requires better knowledge of the genes regulating their synthesis.

Anthocyanins and PAs are two of the six flavonoid subclasses synthesized late in the biosynthetic pathway along with flavonols, flavones, flavanones, and flavan‐3‐ols (monomeric units for PAs) subclasses (Figure S1). These flavonoids are secondary metabolites, synthesized by the flavonoid biosynthetic genes that are grouped into early (EBGs) and late biosynthetic genes (LBGs). The EBGs, which include chalcone synthase, chalcone isomerase, flavanone 3‐hydroxylase, and flavanone 3′‐hydroxylase, are transcriptionally regulated by MYB‐type regulatory proteins (Li, 2014). The LBGs include dihydroflavonol 4‐reductase (DFR), leucoanthocyanidin dioxygenase, anthocyanidin reductase, and TT12, most of which are reported to be activated by a complex known as MBW, formed from three different types of regulatory proteins—a MYB, a beta helix‐loop‐helix (bHLH), and a WD40 (Hichri et al., 2011; Koes et al., 2005; Li et al., 2014). The function of MYB transcription factors (TFs) is to direct the MBW complexes for their differential regulation of the synthesis of flavonoids across plant organs and tissues, while the bHLH TFs regulate the expression of flavonoid biosynthetic genes, sometimes in a partially overlapping pattern involving one or more branches of the biosynthetic pathway (Gonzalez et al., 2008; Kiferle et al., 2015; Montefiori et al., 2015; Petroni & Tonelli, 2011). The WD40 protein stabilizes the MBW complex without a direct regulatory function (Hichri et al., 2011; Ramsay & Glover, 2005).

**Core Ideas**

- Anthocyanins and proanthocyanidins are pigmented flavonoids with desirable health‐promoting properties.
- *Pb* and *Rc* are transcription factors that activate biosynthesis of anthocyanins and proanthocyanidins, respectively.
- Mutual enhancement was discovered, with *Rc* increasing anthocyanin content and *Pb* increasing proanthocyanidins.
- Three QTLs were newly identified as regulating concentrations of anthocyanins and/or proanthocyanidins.
- *Pb*, *Rc*, and these molecularly tagged QTLs can be used to enhance the nutritional quality of rice varieties

The transcriptional regulation of anthocyanins has been well studied in many plant species. In maize, the *C1/Pl* and *R/B* genes encoding R2R3‐MYB and bHLH transcription factors, respectively, coordinately regulate anthocyanin biosynthesis in a tissue‐specific manner (Dooner et al., 1991). The gene *OsC1* in rice, homologous to maize *C1*, has been identified to be a R2R3‐MYB group and is associated with the apiculus anthocyanin pigmentation phenotype (Reddy et al., 1998; Saitoh et al., 2004). Sun et al. (2018) further demonstrated that anthocyanin synthesis in rice hulls was regulated by both the *C1*, a color‐producing gene on chromosome 6 and *S1*, a gene encoding a bHLH protein for tissue specificity for purple phenotype. Conversely, the *C1* MYB TF was not required for pericarp pigmentation (Sun et al., 2018). The mapping of *Kala3* on chromosome 3 and subsequent molecular and genetic analyses demonstrated that it is a MYB TF and a key gene regulating the black (also called purple) rice pericarp trait and the biosynthesis of anthocyanins (Kim et al., 2021; Maeda et al., 2014).

Two bHLH TF genes located on chromosome 4 that regulate rice purple pericarp characteristics were cloned using a cDNA probe of maize *B* gene (Sakamoto et al., 2001). These genes, *OsB1* and *OsB2*, were located 10 kb apart at a *purple leaf* (*Pl*) locus, in which three alleles (*Pl^i^*, *Pl^j^* and *Pl^w^*) were previously identified and shown to control tissue‐specific anthocyanin deposition (Kinoshita & Maekawa, 1986; Sakamoto et al., 2001). Both OsB1 and OsB2 contain conserved N‐terminal acidic domains and bHLH domains, while the C‐terminal domain in OsB1 is completely deleted in OsB2. Either one of them along with maize *C1*, in a transient complementation assay, activate anthocyanin biosynthesis in rice aleurone cells, a cell type that does not normally synthesize anthocyanins (Sakamoto et al., 2001). A *purple bran* (*Pb)* gene controlling the purple pericarp character was mapped to chromosome 4 and sequence analysis revealed that a 2‐bp (GT) deletion in the exon 7 expressed purple rice phenotype while the non‐purple rice lines contained no GT deletion (Wang & Shu, 2007). Today, *OsB1*, *OsPb*, and *OsRa* (a gene homologous to maize *R* gene), all of which were mapped to similar location on chromosome 4 and were found to have striking sequence similarity and exon/intron boundaries, are considered to represent the same gene (Hu et al., 1996, 2000; Sakamoto et al., 2001; Wang & Shu, 2007). Similarly, per Oikawa et al. (2015), *OsB2* is considered the same gene as *OsKala4* reported by Maeda et al. (2014).

Relative to anthocyanins, the factors regulating synthesis of PAs are less investigated. As reported in *Arabidopsis*, PA synthesis is controlled by regulatory proteins forming a MBW complex, similar to anthocyanin regulation (Li et al., 2014). While some of the regulatory proteins are shared between anthocyanins and PAs, such as the WD40 of AtTTG1 and the bHLH of AtTT8, others are specific for PA, such as the MYB of AtTTG2, AtTT1, and AtTT16 (Koes et al., 2005). In wheat (*Triticum asetivum)*, however, only the single *Red grain* (*R)* gene was needed to confer the red pericarp trait (Himi et al., 2005). Currently in rice, PA synthesis has been reported to be regulated by a single gene, *OsRc*, on chromosome 7 (Furukawa et al., 2007; Septiningsih et al., 2003; Sweeney et al., 2006). The *OsRc* is a bHLH TF gene and sequence analysis showed the dominant red allele to be 14 bp longer than the recessive white allele, in which the 14‐bp deletion results in two premature stop codons and a truncated protein before the bHLH domain (Sweeney et al., 2006). Genome‐wide association mapping among pigmented and non‐pigmented rice lines further verified that the *OsRc* and *OsRa* genes were the main effect loci for colored pigments in rice bran (Shao et al., 2011; Xu et al., 2016).

Despite recent advances in knowledge of transcription factors regulating pigmented phenotypes in rice, there is still a large gap in understanding the genetics especially regarding regulation of the variable concentrations of these flavonoids (Poulev et al., 2018). The wide range of concentrations in anthocyanins and PAs of pigmented rice genotypes suggests that concentration differences might be due to genetics as well as environmental effects (Chen et al., 2016, 2017). Tricin is a flavone flavonoid synthesized by the EBGs. In a study of genotypic diversity of tricin content in rice grains, Poulev et al. (2018) demonstrated that purple genotypes (containing anthocyanins) had higher tricin than red and light‐pigmented rice genotypes, indicating that anthocyanin synthesis did not reduce flux through the flavone pathway or vice versa. On the other hand, over expression of anthocyanidin synthase (ANS), also known as leucoanthocyanidin dioxygenase (LDOX), increased the accumulation of a mixture of flavonoids and anthocyanins but lowered the PA content (Reddy et al., 2007). Competition between the flavone and anthocyanin pathways was also demonstrated in T65‐Plw seedlings, a purple‐leaf rice line, relative to T65 (Shih et al., 2008).

We generated a mapping population by crossing a white bran genotype with a pigmented bran genotype known to contain both anthocyanins and PAs to identify QTLs contributing to the concentrations of these pigment‐associated flavonoids. We hypothesized that both structural genes and those encoding regulatory factors might be involved in determining not just the presence/absence of anthocyanins and PAs in rice pericarp, but also their concentrations. Examination of TAC and PA in these segregating progeny lines also allowed us to determine if there is a trade‐off between these two flavonoids that share much of their biosynthetic pathways.

## 2 MATERIALS AND METHODS

### 2.1 Mapping population of F_5_ recombinant inbred lines (RILs)

The mapping population for the study of genetics of anthocyanins and PAs was from a cross between IR36ae x “242.” IR36ae is a white grain rice (*pbpbrcrc*) (Juliano et al., 1990) and “242” (PI 245710) has both anthocyanin (*PbPb*) and proanthocyanidins (*RcRc*) in its pericarp. Molecular markers tagging the *Pb* (anthocyanin synthesis gene, chromosome 4) and *Rc* (PA synthesis, chromosome 7) genes, described in more detail in Section 2.5, were used to verify two F_1_s as true hybrids. F_2_ seed harvested from the two F_1_ plants were seeded into peat pots (6.5 cm × 6.5 cm × 7 cm tall) filled with commercial potting soil (Bacto Premium Potting Soil 0.085‐0.10‐0.05, Michigan Peat Co.) and wetted on May 11, 2016 with fertilizer solution (4 g L^−1^ Jack's Professional 20–20–20 N–P–K [J.R. Peter], plus 0.17 g L^−1^ iron chelate [Sequestrene 330 Fe, 10% Fe, Becker Underwood]) to initiate germination. Plants grew in the greenhouse for 4 weeks, during which time the peat pots were watered regularly (pots were placed on a 2‐cm layer of soil within water‐holding flats) and fertilized twice weekly with the same fertilizer solution. Leaf tissues were analyzed for rice SSR marker RM190 (chromosome 6) in support of research on a trait unrelated to bran color, and on June 8, 2016, all F_2_ plants that proved homozygous for RM190 were transplanted as single plants to the field, of which 428 F_2_ plants survived to produce naturally self‐pollinated seed. For the F_3_ generation, two progeny per F_2_ were grown (total 856 F_3_s), which were advanced by natural self‐pollination and single seed descent to 856 F_2:4_ plants. Grains from F_4_ plants were dehusked and rated visually for having either colored or standard tan (white) bran, and leaf tissues were collected and analyzed per plant for molecular markers tagging *Pb* and *Rc* (described in Section 2.5) that were based on polymerase chain reaction (PCR) amplification.

The F_5_ recombinant inbred lines (RILs) for QTL mapping were enriched for colored‐bran progeny by including in 2019 progeny from just the 418 F_4_ plants having colored bran and confirmed using molecular markers to be homozygous for *Pb*, *Rc*, or both. These F_5_ RILs were germinated as described for the F_2_ generation on April 3, 2019 and transplanted on May 1, 2019 in single‐plant plots using a randomized complete block design with two replications, augmented with eight plots of each parental line per replication. Also transplanted in 2019 were several sets of Pb‐NILs created from F_3_ and F_4_ plants homozygous for *Rc*, but segregating yet for *Pb*. Similarly created Rc‐NILs differing for *Rc* or *rc* were also transplanted, as were F_5_ progeny from F_4_ plants heterozygous at both *Pb* and *Rc*. Grains from F_6_ NILs grown in 2020 were used to study potential trade‐off effects between TAC and PA of combining *Pb* and *Rc*. Leaf tissues were collected from all field‐grown F_5_ and F_6_ plants and analyzed for molecular markers tagging *Pb, Rc*, and a newly identified QTL for the purpose of identifying different subsets of F_5_ plants for the following studies. Characterization of the phenotyping methods for TAC and PA is presented in Sections 2.7 and 2.8, respectively.

### 2.2 Initial mapping of QTL for TAC in 31 *PbPbrcrc* progeny

**FIGURE 1:**
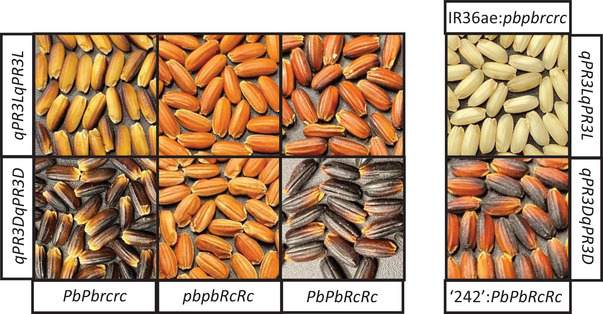
Rice grain color of the genetic variants combining three genes: *Pb*, gene regulates or turns on the synthesis of anthocyanins; *Rc*, gene regulates or turns on the synthesis of proanthocyanidins; and *qPR3*, a quantitative trait locus (QTL) associated with the concentrations of anthocyanins and proanthocyanidins. Photos by Jace Everette, September 2022.

QTLs associated with differences in anthocyanin concentration were mapped in progeny determined from molecular analysis (Section 2.5) to be fixed for *PbPb* to ensure they were producing anthocyanins (Lim & Ha, 2013; Wang & Shu, 2007), and fixed for *rcrc* because TAC is more efficiently measured in the absence of PAs (detailed in Section 2.7). Grains from F_5_ plants determined molecularly to be *PbPbrcrc* were dehusked and rated visually for having light purple or dark purple grains. This was based on visual perception of the color intensity and percentage of the grain area having purple coloration, as can be seen in Figure 1; the two *PbPbrcrc* photos are on the left, with the upper photo showing light purple grain, the lower photo dark purple grain. Visual ratings of the two F_5_ replications were used to identify 15 F_5_ with consistently dark purple bran and 16 having consistently light purple bran. These same two replications of grains were scanned and grain images evaluated for %purpleness as described in Section 2.7. The same grains were also chemically analyzed for TAC and PA, using laboratory methods detailed Sections 2.7 and 2.8, respectively. Genotypic characterization for approximately 1000 single‐nucleotide polymorphism (SNPs) loci is described in Section 2.6.

### 2.3 Additional TAC QTLs sought using *PbPb* progeny homozygous also for *RcRc* and the QTL identified from initial mapping effort

The initial QTL mapping among 31 F_5_ RILs (Section 2.2) identified a QTL for light/dark purple on chromosome 3, molecularly tagged by RM3400. However, when the *PbPbrcrc* F_5_ progeny were further classified for containing or not (+, the “242” allele; ‐, the IR36ae allele) the newly identified QTL on chromosome 3, a wide range of TAC was observed among F_5_ RILs fixed for the “242” allele at RM3400, suggesting the possibility of additional genes. Thus 29 F_5_ RILs now fixed for *RcRc* with *PbPb*, and homozygous also for the “242” allele at RM3400 were genotyped with approximately 1400 SNPs as described in Section 2.6.

### 2.4 QTL mapping of PA in *RcRc* progeny

To assess the regulation of PA, a subset of 76 F_5_ RILs that were molecularly determined to be *RcRc* homozygous was selected for phenotyping and genotyping. Because the presence of anthocyanin does not interfere with measurement of PA, it was not a concern if these F_5_ RILs were homozygous or heterozygous for *Pb* or *pb*. The *RcRc* RILs were genotyped as described in Section 2.6.

### 2.5 PCR‐based molecular markers tagging *Pb*, *Rc*, and *qPR3*

To detect the *Pb* purple pericarp anthocyanin regulatory gene (Rahman et al., 2013), a dinucleotide functional marker was developed based on a 2‐bp (GT) deletion in exon 7 of LOC_Os04g47080. The primer sequences F: ATGCCAGTGGAAGGAATTGC and R:TCCAAACAGACGTATAGATACAGAG amplified a 212 bp fragment in purple pericarp lines and a 214 bp fragment in white lines. The Rid12 marker for the *Rc* gene (LOC_Os07g11020) developed by Sweeney et al. (2006) was used to detect the *Rc/rc* functional nucleotide polymorphism for red pericarp. To fill a SNP marker gap on chromosome 3 in the 1k‐RiCa V1 marker map used to identify the QTL for TAC, all F_5_ and F_6_ RILs were genotyped for six molecular markers that included one marker tagging an insertion/deletion (InDel) site, MJInDel2 (Venu et al., 2014) located at 13,221,636 bp, and five simple sequence repeat (SSR) markers: RM232, RM282, RM15123, RM3400, and RM5626 on chromosome 3 at 9,755,622 bp, 12,408,664 bp, 15,764,172 bp, 17,266,134 bp, and 24,866,271 bp, respectively. The SSR markers were selected using information publicly available at www.gramene.org, where SSR markers and their primers developed by McCouch et al. (2002) are listed by their chromosomal locations along the Os‐Nipponbare‐Reference‐IRGSP‐1.0 assembly (Kawahara et al., 2013). These PCR‐based markers were amplified and analyzed as described in Costanzo et al. (2011).

### 2.6 Genome‐wide SNP characterizations

The initial set of 31 *PbPbrcrc* F_5_ plants were genotyped for some 1000 SNPs using freeze‐dried leaves from the first of the two 2019 field replications and the DNA extraction and genotyping services provided by AgriPlex Genomics (Cleveland, OH) to characterize the plants using the 1K‐Rice Custom Amplicon (1k‐RiCA) (https://doi.org/10.1186/s12284‐019‐0311‐0). A second set of 29 *PbPbrcrc* F_5_ RILs and 76 *RcRc* RILs were characterized by AgriPlex for some 1400 SNPs by using both the IRRI 1k‐RiCA V4 Panel (https://www.agriplexgenomics.com/irri‐v4) (Arbelaez et al., 2019) and the LSU500 panel (Cerioli et al., 2022), which contain some common SNPs.

The SNP calls returned by AgriPlex were manually filtered to remove nonpolymorphic SNPs and SNPs with excessively high failure rates and excessive heterozygosity. Imputation of missing data was accomplished as described in Barnaby et al. (2022) using neighboring marker information and was implemented using the Perl script simple_impute_CSSL.pl (https://github.com/jeremyde/examples_and_templates/blob/master/simple_impute_CSSL.pl). Heterozygous allele calls were converted to missing data to conform to a RIL population QTL model.

### 2.7 Analysis of grains for TAC and purpleness

Previous study of 42 purple pericarp cultivars showed that TAC was associated with color intensity determined by a colorimeter (Chen et al., 2017), suggesting that visual ratings and measurement of purple intensity using image analysis would be non‐destructive methods for mapping TAC. Grains of both 2019 replications of the 31 RILs selected as per Figure 1 *PbPbrcrc* photos for their dark and light purple extremes were scanned with an imaging analysis system (WinSeedle™ Pro version 2013b, Regent Instruments, Canada Inc., Sainte‐Foy Quebec, Canada) and quantified for their percentage of grain surface area comprised of dark (purple) pixels, a trait now designated as “%purpleness.”

For analysis of TAC and PA, grains were first ground to flour for 2 min using Mini‐Beadbeater‐96 with stainless tube and beads (BioSpec Products, Inc., Bartlesville, OK, USA). Then, for RILs not containing PAs (molecularly *rcrc*), a subsample of 100 mg whole grain flour was extracted with 0.8 mL acidified methanol (85% methanol in 0.23 N HCl) for 1 h at room temperature with shaking; the mixture was centrifuged for 10 min at 12.4 K × *g* and the supernatant was saved. The pellet was extracted one more time with 0.8 mL acidified methanol. The pooled supernatant was used for anthocyanins analysis (Min et al., 2011).

The concentration of anthocyanins in the extract was determined using a microplate reader by measuring the absorbance at wavelength 530 nm. The absorbance of the sample extract was then corrected for its turbidity at wavelength 700 nm. This was followed by background noise subtraction at wavelength 530 nm using extract of IR36ae after subtracting its absorbance at 700 nm for turbidity. After the corrections for turbidity and background noise, TAC in the extract was calculated against a linear curve of kuromanin (cyanidin‐3‐glucoside chloride, Sigma‐Aldrich, St. Louis, MO., USA). For RILs known to contain PA (molecularly *RcRc or Rcrc*), the extract was first adsorbed with Sephadex LH‐20 (Lipophilic Sephadex, Sigma‐Aldrich, St. Louis, MO., USA) to remove interfering compounds near 450 nm prior to the microplate spectrophotometric analysis. Briefly, 100 mg LH‐20 was hydrated in 0.4 mL 50% methanol in a 2‐mL tube, and then a 1‐mL sample extract, which was first equilibrated to be in 75% methanol using 100% MilliQ water, was added. After 30 min of gentle shaking, the tube was centrifuged for 1 min and the supernatant was measured at 520 nm for anthocyanins. After the subtraction of the turbidity and background noise, the extract absorbance was multiplied by a conversion factor of 1.9, which was then used to calculate the anthocyanin concentration against the linear curve of kuromanin. The LH‐20 adsorption method was validated using six sets of extracts not containing PAs, and the TAC value of the extract adsorbed with LH‐20 was 104 ± 7% of that without LH‐20 adsorption. For the Rc‐NIL study, which segregated *PbPbRcRc* and *PbPbrcrc*, all the sample extracts were adsorbed with LH‐20 gel prior to the spectrophotometric analysis and quantification.

### 2.8 Analysis of grains for PA

PA was determined using the 4‐dimethylaminocinamaldeyde (DMAC) method of Prior et al. (2010) with some modification. Briefly, 100 mg of whole grain flour was extracted with 0.8 mL acidified methanol (85% methanol in 0.23N HCl) for 1 h at room temperature with shaking; the mixture was centrifuged for 10 min at 12.4 K × *g* and the supernatant was saved. The extraction was repeated one more time. The pooled supernatant was used for PA analysis. For the DMAC color reaction, an aliquot of 50 μL extract and 150 μL of 0.1% DMAC was added to a 96‐well microplate and the color reaction was read every 1.5 min for 45 min, at 25°C. The maximum absorbance of the sample extract and the standards (procyanidin B2, Sigma‐Aldrich, St. Louis, MO., USA) was used to calculate the PA in the extract. Presence of anthocyanins does not interfere with the DMAC method for determining PA.

### 2.9 Grain trait statistics

Summary statistics, frequency distribution, analysis of variance (ANOVA), and Fisher's least significant difference (LSD) mean comparison for the TAC and PA were performed in Minitab® statistical software (Minitab Ltd., Coventry, UK). JMP 14 (SAS Institute Inc. 2018) was used to calculate best linear unbiased predictors (BLUPs) from multiple replications and years of trait data for use in QTL analyses.

### 2.10 QTL analyses

Identification of QTLs based on non‐random distribution of SNP alleles between the qualitative light purple versus dark purple classifications from the 31 *PbPbrcrc* plants, genotyped with the 1k‐RiCA V1 SNP panel, used the R/qtl2 package (Broman et al., 2019). The grain color classification data were coded as 0 for light purple and 1 for dark purple. Log‐of‐odds (LOD) thresholds were established for α = 0.1 and α = 0.05 using 1000 permutations. The locations of significant QTL peaks were established using the LOD thresholds and 95% Bayesian credible intervals.

QTL analyses for %purpleness and TAC in these 31 RILs, and QTL mapping of TAC and PA in the additional populations genotyped with the 1k‐RiCA V4 and LSU500 SNP datasets were conducted using the single marker regression (SMR), composite interval mapping (CIM LS), and multiple interval mapping (MIM) modules in QGene V4.4.0 (Joehanes & Nelson, 2008). Heterozygous allele calls were converted to missing data to conform to the RIL population QTL model in QGene. LOD thresholds at alpha = 0.1 and alpha = 0.05 were determined per trait using 1000 permutations. The left and right edges of QTLs were defined as regions having a LOD score greater than peak LOD minus 1, or the LOD threshold, whichever was higher. Tables, figures, and discussion text will focus on CIM results and include MIM when considering multiple peaks per trait. QTLs were named according to the nomenclature guidelines by McCouch (2008), with a “q,” followed by trait acronym and number indicating the chromosome onto which the QTL mapped.

## 3 RESULTS

### 3.1 Summary of TAC and PA of RILs

The quantitative phenotypic traits of the subset of 31 F_5_ RILs visually selected for having extreme dark (*n* = 15) and light purple (*n* = 16) grains verified using molecular markers (Pb and Rid12) to be *PbPbrcrc* are presented in Table 1, with example samples presented in Figure 1. The average TAC for “Dark” and “Light” purple classes were 352.8 and 16.8 μg g^−1^, respectively. The average “%purpleness” of pixel purple area per kernel for “Dark” and “Light” were 51.5% and 12.3%, respectively. The average PA in *RcRc* lines (*n* = 76) was 1147.2 and 1025.0 μg g^−1^ in F_5_ and F_6_, respectively. The ranges of PA were more than 7x in F_5_ and > 9x in F_6_.

**TABLE 1:** Phenotypic traits^a^ of purple and red grains of selected RILs for QTL mapping

Grain characteristics	# RILs	Genotype	TAC (μg g^−1^)			%Purpleness (%)^b^			PA (μg g^−1^)		
			Mean	Min	Max	Mean	Min	Max	Mean	Min	Max
Extreme dark purple	F_5_, *n* = 15	*PbPbrcrc*	352.8	39.5	926.6	51.5	38.1	71.9	NA	NA	NA
Extreme light purple	F_5_, *n* = 16	*PbPbrcrc*	16.8	7.2	41.9	12.3	5.9	18.9	NA	NA	NA
Red	F_5_, *n* = 75	*RcRc*	NA	NA	NA	NA	NA	NA	1147.2	351.7	2457.6
Red	F_6_, *n* = 75	*RcRc*	NA	NA	NA	NA	NA	NA	1025.0	294.8	2861.3

Abbreviations: PA, total proanthocyanidin content; QTL, quantitative trait locus; RIL, recombinant inbred line; TAC, total anthocyanin content.

^a^ NA indicates that trait was not analyzed in the listed genotypes.

^b^ Percentage of grain area in scanned image having dark (purple) coloration.

The purple grains of 147 RILs (Pb‐RILs) of F_5_ (2019) and F_6_ (2020) having *PbPbrcrc* and *PbPbRcRc* were used for validating and molecularly tagging a QTL for TAC using PCR‐based molecular markers. The TAC in grains of F_5_ and F_6_ RILs having *PbPb* were on average 127.2 and 184.2 μg g^−1^, respectively. The TAC in grains from some F_5_ Pb‐RILs was below the minimum detection level (expressed as 0 μg g^−1^), while the minimum F_6_ TAC was 4.7 μg g^−1^. The maximum TAC for F_5_ and F_6_ RILs’ grains were 730.5 and 1545.4 μg g^−1^, respectively. The Pb‐RIL extremes were lower and higher than the TAC of “242” pigmented grains (1144.1 and 974.0 μg g^−1^) in 2019 and 2020, respectively. The averaged TAC for the Pb‐RILs was slightly lower than the averaged TAC (184.8 μg g^−1^) from the subset of extreme dark/light RILs plus “242” parent used for QTL mapping.

The grains of 176 RILs (Rc‐RILs) having *pbpbRcRc* and *PbPbRcRc* were used for validating and molecularly tagging a QTL for PA using PCR‐based molecular markers. The averaged PA in seeds of F_5_ and F_6_ were 987.9 and 865.1 μg g^−1^, respectively. The minimum PA in F_5_ and F_6_ were 310.5 and 466.6 μg g^−1^, respectively, while the maximum PA in F_5_ and F_6_ were 2061.5 and 3146.8 μg g^−1^, respectively, which were lower and higher than the PA of “242” pigmented grains (2223.8 μg g^−1^ in 2019 and 1796.6 μg g^−1^ in 2020). Similar ranges of PA (6.6×) were found between F_5_ 176 Rc‐RILs and the QTL mapping subset. However, a wider range of PA in F_6_ Rc‐RILs was shown (17×) relative to the randomly selected subset of RILs used for PA QTL mapping.

Analyses of variances of TAC and PA indicated that the genotypic factor of “RILs” accounted for most of the variances of TAC (*R^2^* = 0.76) and PA (*R^2^* = 0.84), while “Year” (F_5_ and F_6_ generations in 2019 and 2020, respectively) explained significant but small proportions of variation that were less than 1/10^th^ and 1/20^th^ the TAC and PA variations explained by RILs in each year.

### 3.2 QTLs for TAC and color among 31 *PbPbrcrc* RILs with extreme color phenotypes

Among the 1k‐RiCA V1 SNPs, 446 were polymorphic between IR36ae and “242,” but with the small population size (*n* = 31) restricting recombination rates, several closely linked SNPs mapped to the same cM location in the genetic map, making them redundant. A total of 259 non‐redundant polymorphic SNPs from the 1k‐RiCA V1 Panel were used for our marker‐trait analyses. The average cM distance between non‐redundant marker loci in the genetic map was 4.7 cM, with two large gaps: a 47.2 cM gap on chromosome 2 and a gap greater than 50 cM on chromosome 3 (Figure S2). The marker gap on chromosome 3, from 03_14933451 to 03_31427789, proved especially problematic because the QTL analyses of both color class and %purpleness identified a single QTL on chromosome 3 with its peak at 03_14933451 (Table 2; Figure S2). These were large QTLs that spanned the marker gap but showed stronger linkage to 03_14933451 than to 03_31427789. On the other hand, analysis of the TAC data from grains harvested in 2019 indicated two QTLs, one on chr3 having the same QTL peak and span as the color class and %purpleness QTLs, which we now name as *qPR3*, and a second QTL on chr7 (*qPR7*) between 07_19408599 and 07_23535052.

**TABLE 2:** QTLs identified by interval mapping as associated with rice pigmented pericarp traits in segregating progeny from a cross between IR36ae (white pericarp) x “242” (purple and red pericarp)

QTL	Trait^a^, number and type of RILs studied, and year(s) of grain harvest with 2 replications per year	Peak SNP location (Chr_bp)^b^	Left flank SNP (Chr_bp)^b^	Right flank SNP (Chr_bp)^b^	LOD^c^	Additive effect^d^	Percentage variance explained	Candidate gene^e^
*qPR3*	Dark/Light purple class, 31 *PbPbrcrc* RILs, grain harvested 2019	03_14933451	03_12564300	03_31427789	4.49**	na	48.3%	LOC_Os03g29614, LOC_Os03g32470
*qPR3*	%purpleness, 31 *PbPbrcrc* RILs, grain harvested 2019	03_14933451	03_12564300	03_31427789	4.499**	14	43.8%	
*qPR3*	TAC, 31 *PbPbrcrc* RILs, grain harvested 2019	03_14933451	03_12564300	03_31427789	3.523*	132	36.3%	
*qPR3*	PA, 75 *RcRc* RILs, grain harvested 2019	03_16734121	03_15923846	03_23010799	9.332**	330	42.0%	
*qPR3*	PA, 75 *RcRc* RILs, grain harvested 2020	03_21502680	03_15923846	03_23010799	9.014**	318	40.9%	
*qPR3*	PA, 75 *RcRc* RILs, 2‐year BLUP	03_16734121	03_15923846	03_23010799	10.53**	299	45.9%	
*qPR5*	PA, 75 *RcRc* RILs, grain harvested 2019	05_18005952	05_18005952	05_18405433	3.932*	177	20.5%	No candidate in QTL
*qPR5*	PA, 75 *RcRc* RILs, grain harvested 2020	05_18405433	05_18005952	05_18405433	3.324 ns	151	17.6%	
*qPR5*	PA, 75 *RcRc* RILs, 2‐year BLUP	05_18005952	05_18005952	05_18405433	4.016*	149	20.9%	
*qPR7*	TAC, 31 *PbPbrcrc* RILs, grain harvested 2019	07_19408599	07_18689329	07_23535052	3.376*^f^	‐41	35.1%	LOC_Os07g32620

Abbreviations: LOD, log‐of‐odds; PA, total proanthocyanidin content; QTL, quantitative trait locus; RIL, recombinant inbred line; SNP, single‐nucleotide polymorphism; TAC, total anthocyanin content.

^a^ Dark/light purple class, visual classification of grains into dark or light purple based on both the color intensity and proportion of surface area having purple or black coloration; %purpleness, percentage of the pixels in the grain surface area in a scanned image having dark (purple) coloration.

^b^ *Oryza sativa* SNPs are identified by their physical location (base pair, bp) based on the Os‐Nipponbare‐Reference‐IRGSP‐1.0 assembly(MSU7) (Kawahara et al., 2013).

^c^ *, ** indicate LOD scores were significant at the α = 0.1 or 0.05 level, respectively, and ns indicates not significant.

^d^ A positive allele effect reflects more intense coloration associated with the “242” allele.

^e^ Candidate gene loci are names as they are as listed in MSU7.

^f^ *qPR7* acquired significant LOD during single marker regression (SMR), but not with composite interval analysis (CIM LS) in QGene V4.4.0.

To confirm and more precisely map *qPR3*, molecular markers surrounding this chr3 locus were used to genotype F_5_ and F_6_ Pb‐RILs (*n* = 147). This analysis used TAC data from grains harvested in two years (2019 F_4:5_ and 2020 F_4:6_), two replications per year. The five SSRs and the InDel ranged from chr3 9,755,622 to 24,866,271 bp (Table 3). The markers found to be most closely associated with TAC were RM3400 (03_17266134 bp) (*R^2^* = 0.419 and 0.308 in F_5_ and F_6_, respectively) and RM15123 (03_15764172) (*R^2^* = 0.360 and 0.274 in F_5_ and F_6_, respectively). This verified *qPR3* as a TAC QTL, mapped it more precisely between RM15123 (03_15764172) and RM5626 (03_24866271), and showed that it could be molecularly tagged in segregating progeny using RM3400. The “242” allele at RM3400 was associated with dark purple, while the IR36ae allele was associated with lighter purple. Throughout this manuscript we will distinguish the dark and light alleles as RM3400D and RM3400L, and consider these markers to indicate presence of the *qPR3D* dark or *qPR3L* light alleles, respectively.

**TABLE 3:** Percentage of variance explained (from ANOVA *R^2^*)^a^ by polymerase chain reaction (PCR)‐based molecular markers for total anthocyanin content (TAC) in Pb‐RILs or for total proanthocyanidin content (PA) in Rc‐RILs

SSR/InDel marker	Chr	Physical location^b^	TAC in Pb‐RILs (* n* = 147)^c^		PA in Rc‐RILs (n = 176)^d^	
			F_5_	F_6_	F_5_	F_6_
RM232	3	9,755,622	ns	5.8%	18.7%	12.1%
RM282	3	12,408,664	12.6%	13.8%	21.5%	14,7%
MJInDel2	3	13,221,636	25.2%	18.5%	25.2%	19.6%
RM15123	3	15,764,172	36.0%	27.4%	30.5%	29.1%
RM3400	3	17,266,134	41.9%	30.8%	38.2%	33.3%
RM5626	3	24,866,271	10.8%	8.9%	10.9%	12.1%
Pb	4	27,949,384	NA	NA	ns	3.8%
Rid12	7	6,062,889	4.1%	ns	NA	NA

Abbreviations: RIL, recombinant inbred line.

^a^ NA, trait not applicable to the study population; ns, marker was not significant (*p* > 0.05) in that model analysis.

^b^ Physical locations (in base pairs, bp) of molecular markers are as reported at www.gramene.org and based on Nipponbare (MSU7) (Kawahara et al., 2013).

^c^ Pb‐RILs, RILs homozygous for *PbPb*.

^d^ Rc‐RILs, RILs homoygous for *RcRc*.

The chromosome 7 QTL peak mapped between SNPs at 07_19408599 and 07_23535052, within a 38 cM marker gap. This QTL region contains just one gene known to be associated with color pigmentation, *anthocyanidin 3‐O‐glucosyltransferase* (*AGT*) (LOC_Os07g32620) (Cohen 2019). Evidence of *qPR7* was less confident than that for *qPR3*. Interval mapping placed both QTL peaks in marker gaps, but while *qPR3* had significantly associated markers on both sides of the marker interval, for *qPR7* only one of the flanking markers (07_19408599) was significantly associated with TAC, and it was the allele from unpigmented IR36ae that was associated with increased TAC. Furthermore, analysis of the TAC data using MIM retained a notable QTL peak for *qPR3*, but not for *qPR7* (Figure S2) suggesting it might be a false positive from pseudolinkage within our small population (*n* = 31) of extreme phenotypes. The putative *qPR7* QTL was examined in an additional study (Section 3.3).

### 3.3 Additional anthocyanin QTLs sought using RILs fixed for *Pb*, *Rc*, and *qPR3D* and a denser genetic map

Segregation for loci of large effect can confound ability to identify other QTLs. Therefore, a second effort to identify TAC QTLs involved 29 RILs that were fixed for *PbPbRcRc*, and fixed as well for *qPR3D*, the “242” allele. To increase reliability of TAC data for QTL mapping, these RILs were evaluated using two years (F_4:5_ 2019, F_5:6_ 2020) of grains, two replications per year, and QTL analyses were run using a 2‐year BLUP calculated across years, as well as with single‐year BLUPs. RILs were genotyped by AgriPlex using both the 1K RiCA V4 and the LSU500 panel. This generated 1574 SNP datapoints per genotype, but due to overlap between SNP of the two panels, there were approximately 1400 unique SNPs represented, 528 of which were polymorphic between IR36ae and “242.” When analyzed among the total 92 F_5_ RILs selected for TAC and PA analysis, they yielded a genetic map containing 401 discreet SNP loci separated by an average 2.7cM, with only three marker‐gaps greater than 20 cM (on chr 1, 2, 2), and no marker gaps exceeding 50cM (Figures S2d and S3a–c). Interval analysis of the 2019, 2020, and 2‐year TAC BLUPs were in consensus finding no additional QTLs. Hence, this analysis of 29 RILs fixed as *PbPbRcRcqPR3DqPR3D* was unable to provide further evidence for *qPR7*.

### 3.4 QTL of PA

A total of 75 F_5_ RILs having *RcRc* were randomly selected for mapping PA QTLs. Two QTLs were identified, one on chromosome 3 and another on chromosome 5 (Table 2; Figure S3). The peaks of the PA QTLs differed slightly between 2019, 2020, and the 2‐year BLUP data, but there was consensus in placing a QTL on chr3 with its peak between 15.4 and 21.5 Mb, making it co‐located with the *qPR3* QTL identified as impacting dark/light purple classification, %purpleness, and TAC. This PA QTL had LODs > 8 in all datasets, explained from 40% to 45% of the variance in PA, with an additive effect of 295 units or more. The same six PCR‐based markers on chr3 used to validate *qPR3* for TAC among a larger set of *PbPb* RILs (Section 3.2) also validated association with PA within a larger set (*n* = 176) of *RcRc* RILs (Table 3). Of the six PCR‐based markers, RM3400 (*R^2^* = 0.382 and 0.333 in F_5_ and F_6_, respectively) and RM15123 (*R^2^* = 0.305 and 0.291 in F_5_ and F_6_, respectively) were the markers having the highest *R^2^* values explaining PA (Table 3).

The SNPs flanking the PA QTL peak on chromosome 3 in both the 2020 and 2‐year BLUP analyses were 03_21003799 and 03_21502680, while the 2019 data placed the QTL peak between 03_16734121 and 03_17794287. These peaks encompass or are near RM3400 at 03_17266134. Altogether, the data indicate that the PA QTL on chromosome 3 is the same as *qPR3*, first identified for association with TAC and purple colorations. In fact, this locus was named *qPR3* to indicate its association with both purple and red coloration.

The second PA QTL, *qPR5*, achieved a significant LOD in the F_6_ (2020) and 2‐yr BLUP data analyses, but a subthreshold LOD peak in analysis of F_5_ (2019) data (Table 2; Figure S3). There was consensus among all three datasets in placing *qPR5* between the SNPs 05_18005952 and 05_18405433. *qPR5* had smaller genetic effect than *qPR3* all three datasets, with additive effects and percentages of variance explained approximately half those for *qPR3*. None of the genes between 05_18005952 and 05_18405433 had annotated gene functions associating them with flavonoid concentration, preventing identification of a candidate gene within *qPR5*. This QTL region is, however, flanked by a *MYB TF* at 19.0 Mb (LOC_Os05g04210) and a *WRKY* gene at 17.4 Mb (LOC_Os05g03900). WRKY TFs were shown to affect MYB‐regulation of anthocyanin and/or proanthocyanidin biosynthesis and vacuolar transport in apples (An et al., 2019; Mao et al., 2021), pears (Alabd et al., 2022; Li et al., 2020), and grapes (Amato et al., 2019).

### 3.5 Means and ranges of TAC in Pb‐RILs having *qPR3D* versus *qPR3L*

**FIGURE 2:**
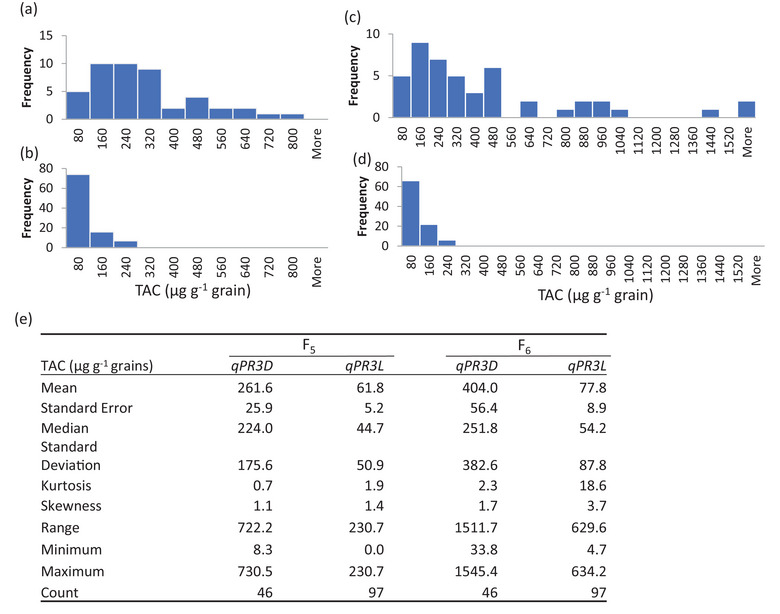
Frequency distributions of total anthocyanin content (TAC) (μg g^−1^ grain) of F_5_ and F_6_ Pb‐RILs homozygous for the *qPR3D* allele (a,c) and those homozygous for the *qPR3L* allele (b,d) are shown. Summary statistics are presented in (e). RIL, recombinant inbred line.

Frequency distributions and descriptive statistics of TAC in F_5_ and F_6_ Pb‐RILs divided by containment of *qPR3D* and *qPR3L* alleles are presented (Figure 2). The Pb‐RILs with *qPR3D* (*n* = 46) had a mean TAC of 261.6 and 404.0 μg g^−1^ in F_5_ and F_6_, and were > 4.2x and > 5.1x of those of RILs having *qPR3L* (61.8 and 77.8 μg g^−1^), respectively. The ranges were also very different with the range of TAC in Pb‐RILs having *qPR3D* being 3.1x and 2.4x of that of q*PR3L* Pb‐RILs in F_5_ and F_6_, respectively. However, the results clearly showed that Pb‐RILs with *qPR3L* deposited low amounts of anthocyanins in the pericarp of pigmented grains, indicating reduced but not entire inactivation of anthocyanin synthesis.

### 3.6 Means and ranges of PA in Rc‐RILs having *qPR3D* versus *qPR3L*

**FIGURE 3:**
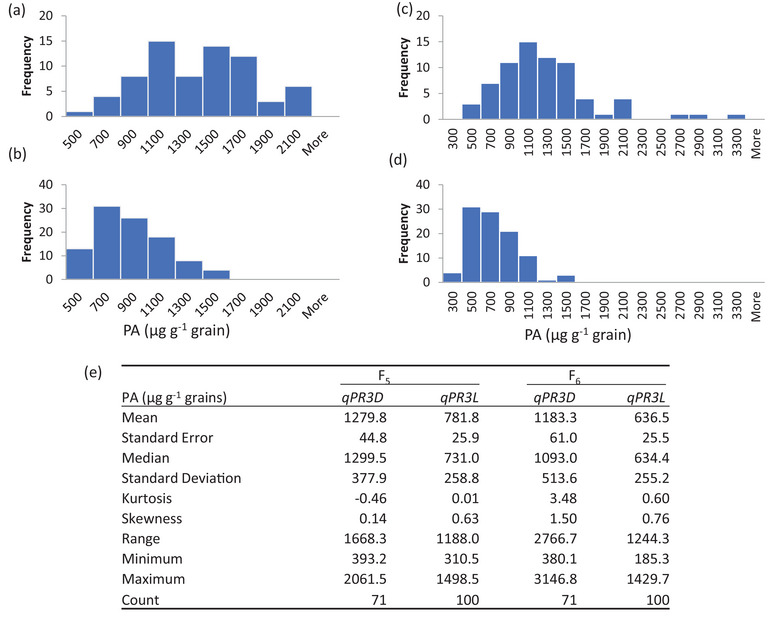
Frequency distributions of total proanthocyanidin content (PA) (μg g^−1^ grain) of F_5_ and F_6_ Rc‐RILs homozygous for the *qPR3D* allele (a,c) and those homozygous for the *qPR3L* allele (b,d) are shown. Summary statistics are presented in (e). RIL, recombinant inbred line.

Frequency distributions and descriptive statistics of PA in Rc‐RILs divided by *qPR3D* and *qPR3L* are presented (Figure 3). The Rc‐RILs with *qPR3D* had wider ranges, higher means, and higher extreme PA phenotypes than Rc‐RILs with *qPR3L*. Rc‐RILs with *qPR3D* (*n* = 71) had average PA of 1279.8 and 1183 μg g^−1^ in F_5_ and F_6_, respectively. Rc‐RILs with *qPR3L* (*n* = 100) had average PA of 781.8 μg g^−1^ in F_5_ and 636.5 μg g^−1^ in F_6_. The PA in Rc‐RILs with *qPR3D* was 64% and 86% higher than that in Rc‐RILs with *qPR3L* in the F_5_ and F_6_ generations, respectively.

### 3.7 Effect of *Rc* and *qPR3* on TAC

The synthesis of anthocyanins and PAs shares the entire portion of the flavonoid biosynthetic pathway that is controlled by EBGs (*CHI, CHS, F3H, F3’H*) and *DFR*, a LBG, and these genes are expressed in grains having both red and purple pericarps (Li et al., 2014; Lim & Ha, 2013; Oikawa et al., 2015). Here we assessed if active versus inactive Rc TF, known to turn on or off production of PAs, would have effect on TAC. This was not addressed during the TAC QTL mapping efforts because the first mapping population was fixed as *PbPbrcrc* and the second fixed as *PbPbRcRc*, with neither population segregating for *Rc/rc* as needed to address the question. Using Rid12 to distinguish RILs as having active (*Rc*) or inactive (*rc*) Rc, and using RM3400D versus RM3400L to distinguish RILs having the *qPR3* allele for darker or lighter colors, we evaluated the effects of +/− *Rc* and *+/*− *qPR3* within the 147 Pb‐RILs. ANOVA of F_5_ data showed that +/− *qPR3* significantly impacted TAC (*p* < 0.001) while the effect of Rid12 was significant at *p* = 0.058; for F_6_ TAC data, only *qPR3* was significant (*p* < 0.001) while *Rc* was not (*p* = 0.77). Dividing the Pb‐RILs based on +/− *Rc* and +/− *qPR3* creates four genetic classes. In agreement with the QTL mapping, Pb‐RILs homozygous for *qPR3D* contained more TAC than those with *qPR3L* (Figure S4). Therefore, it was important to look at +/− *Rc* effects in Pb‐RIL classes also fixed for *qPR3*. Within Pb‐RILs homozygous for *qPR3D*, the TAC did not differ for +/− Rc. In contrast, among RILs having *qPR3L*, the *rcrc* RILs had lower average TAC than *RcRC* RILs in both F_5_ (2019) and F_6_ (2020) data, though the means were significantly different only in the F_5._

**FIGURE 4:**
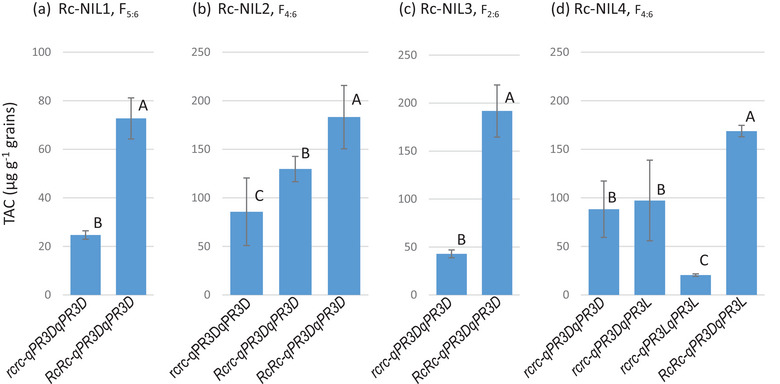
The effects of *Rc* and *qPR3* (as tagged by RM3400) on total anthocyanin content (TAC, μg g^−1^ grain) using Rc‐NILs having *PbPb*. (a–c) F_6_ Rc‐NILs, progeny lines from individual *PbPbRcrc*‐RILs of F_5_, F_4_, and F_2_ plants, respectively, now segregating for *rc* and *Rc*. (d) Rc‐NIL4 are progeny lines from an F_4_ that was heterozygous for both *Rc* and *qPR3*. Different letters for the means indicate significant difference among the genetic variants at 95% confidence level using Fisher LSD method. LSD, least significant difference; NIL, near isogenic line; RIL, recombinant inbred line.

Visible in Figure S4, and also in Figure 2a,c, the Pb‐RILs containing *qPR3D* had especially wide ranges in TAC. Along with the high standard deviations seen for all four genetic classes in Figure S4, the data suggest that unidentified factors are causing wide variance for TAC among the 18 to 51 individuals per genetic class, and this wide variance could be masking any smaller variance attributable to +/− *Rc*. To minimize the possible confounding effects from segregation of other genes, we then used residual heterozygosity for *Rc* found within some of the RILs to create NILs +/− *Rc* (hereafter called Rc‐NILs) with which to evaluate effect of *Rc* in pairs of more uniform genetic backgrounds (Figure 4).

All four sets of Rc‐NILs are F_6_ generation and are homozygous for the *Pb* and *qPR3D* (Figure 4a–c). The Rc‐NIL1 set contains *RcRc* and *rcrc* progeny from an *Rcrc* F_5_ line that was fixed for both *Pb* and *qPR3D*. The TAC averaged over multiple *RcRc* F_6_ plants (TAC = 72.7 μg g^−1^) was 2.9x of that of the *rcrc* sibling NILs (TAC = 24.7 μg g^−1^). Rc‐NIL2 was a set of homozygous and heterozygous F_6_ progeny lines descendent from a heterozygous *Rcrc* F_4_ plant, and the averaged TAC of F_6_ Rc‐NIL2 plants having *RcRc* (183.2 μg g^−1^) and *Rcrc* (129.7 μg g^−1^) were 2.1x and 1.5x of the TAC in *rcrc* NIL progeny (85.7 μg g^−1^), respectively. The Rc‐NIL3 were F_6_ progeny descendent from divergence for *RcRc* versus *rcrc* detected in the F_3_ generation (traced to same F_2_ parent), and the averaged TAC of *RcRc* (191.8 μg g^−1^) was 4.5x of that of rcrc (42.8 μg g^−1^). These three sets of Rc‐NILs all indicated that synthesis of PA from *Rc* caused an increase in TAC.

Rc‐NIL4 consisted of F_6_ progeny from an F_4_ fixed for *Pb* but heterozygous for both *Rc* and *qPR3* (Figure 4d). The TAC of *rcrc* F_6_‐NILs homozygous also for *qPR3D* (88.3 μg g^−1^) were not different from those heterozygous at *qPR3* (97.3 μg g^−1^), but both had significantly higher TAC than *rcrc* NILs having *qPR3L* (20.4 μg g^−1^), indicating that *qPR3D* has complete dominance over *qPR3L*. For evaluation of Rc effects, progeny in this NIL set that were *RcRc* and heterozygous for *qPR3* had the highest averaged TAC (168.8 μg g^−1^) among the four progeny genotypes, higher than any of the *rcrc* progeny, providing further evidence that *Rc* positively impacts TAC.

### 3.8 Effect of *Pb* and *qPR3* on PA

The QTL mapping among Rc‐RILs detected an effect of *qPR3* on PA but did not detect an effect from *Pb*, though it also segregated in this relatively small population (*n* = 75). The effects of *Pb* and *qPR3* on PA were investigated further using 170 Rc‐RILs evaluated for PA over 2 years, the F_5_ and F_6_ generations. The ANOVA detected both *Pb* and *qPR3* as significant factors in the model, explaining PA at *R^2^* = 0.014 (*p* = 0.052) and *R^2^* = 0.38 (*p* < 0.001), respectively, in F_5_ Rc‐RILs and at *R^2^* = 0.041 (*p* = 0.001) and *R^2^* = 0.33 (*p* < 0.001), respectively, in F_6_ Rc‐RILs. Thus, though Pb effects were found significant, they explained 1/10^th^ the variance in PA as that explained by *qPR3*. Use of means comparison to compare the average PA among the four genetic classes within the Rc‐RILs (Figure S5) showed that Rc‐RILs containing *qPR3D* had more PA than those with *qPR3L*, and this was true in both the F_5_ and F_6_ generations, regardless of whether the Rc‐RILs contained *Pb* or *pb*. In contrast, effects of *Pb* on PA were seen only among F_6_ Rc‐RILs homozygous for *qPR3D*, where Rc‐RILs homozygous for *Pb* had more PA than those homozygous for *pb*. The effect of *Pb* was not significant among grains harvested from F_5_ Rc‐RILs in 2019, nor was it significant among the F_6_ Rc‐RILs homozygous for *qPR3L*.

**FIGURE 5:**
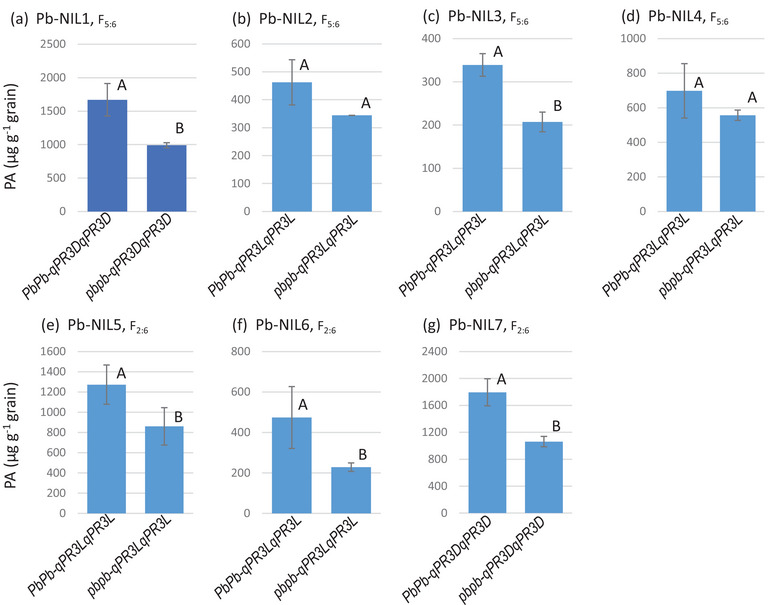
The effects of *Pb* and *qPR3* (as tagged by RM3400) on total proanthocyanin content (PA, μg g^−1^ grain) using Pb‐NILs having *RcRc* and fixed also for either the *qPR3D* or *qPR3L* alleles from the “242” or IR36ae parents, respectively. Pb‐NILs 1 through 3 (a–c) are F_6_ Pb‐NILs, progeny lines from a *Pbpb* F_5_ RIL; PB‐NIL4 (d) is a set of F_6_ progeny differing for *PbPb* or *pbpb* and derived from a single F_4_ RIL. (e–g) F_6_ Pb‐NILs differing for *PbPb* or *pbpb* due to divergence that occurred in the F_2:3_ generation. Different letters for the means indicate significant difference among the genetic variants at 95% confidence level using Fisher LSD method. LSD, least significant difference; NIL, near isogenic line; RIL, recombinant inbred line.

However, the wide variability for PA within each genotypic class of the 76 Rc‐RILs (Figure S5) can make it difficult to detect a small effect of *Pb* on PA. To reduce the background noise, PA was further studied using Pb‐NILs, paired F_6_ progeny differing only for *Pb/pb* because they were fixed in a previous generation for *Rc* and *qPR3* (Figure 5). Pb‐NIL sets 1 through 3 (Figure 5a–c) diverged for *Pb* and *pb* in the F_5_ generation, Pb‐NIL4 (Figure 5d) diverged in the F_4_ generation, and the remaining three Pb‐NIL sets (Figure 5d–f) each traced to a different F_2_ parent, and F_3_ divergence for *Pb/pb*. For all seven Pb‐NIL paired sets, the multiple *PbPb* progeny had more PA than the *pbpb* progeny, though this difference was not significant for two of the seven sets (Pb‐NIL2 and Pb‐NIL4). The PA averages in the *PbPb* versus *pbpb* progeny were, from Pb‐NIL 1 through 7 (Figure 5a–g), 1670 μg g^−1^ versus 990 μg g^1^, 462 μg g^−1^ versus 344 μg g^−1^, 339 μg g^−1^ versus 207 μg g^−1^, 698 μg g^−1^ versus 557 μg g^−1^, 1272 μg g^−1^ versus 861 μg g^−1^, 474 μg g^−1^ versus 229 μg g^−1^, and 1794 μg g^−1^ versus 1062 μg g^−1^, respectively. These seven *PbPb* versus *pbpb* comparisons among related progeny lines demonstrated that the Pb TF positively modulates PA.

## 4 DISCUSSION

In this study, we identified three factors affecting TAC after activation of anthocyanin biosynthesis by *Pb*, namely *qPR3, qPR7*, and the *Rc* TF, and identified three factors affecting PA after activation of PA biosynthesis by *Rc*, specifically *qPR3, qPR5*, and the *Pb* TF. These factors regulating the concentrations of pigmented flavonoids (anthocyanins and PAs) were identified using RILs with pigmented grains from an IR36ae x “242” mapping population. IR36ae has white pericarp, and “242” is a pigmented rice cultivar containing both anthocyanins and PAs and was ranked among the highest of 25 purple rice genotypes for its total flavonoid and phenolic contents as well as antioxidant capacity (Chen et al., 2017; Wan et al., 2021).

Effect of *qPR3* on TAC was verified with independent identification in two population subsets (*PbPbrcrc, n* = 29, and *PbPbRcRc, n* = 31), documented also in a larger set of 147 Pb‐RILs genotyped using SSRs tagging *qPR3*, and further validated in a set of NILs (Rc‐NIL4). Effect of *qPR3* on PA was also verified with identification in two populations, being detected initially in 76 SNP‐genotyped Rc‐RILs, then validated in a set of 176 SSR‐genotyped Rc‐RILs. The effect of *Pb* on PA is smaller than that of *qPR3* and was not detectable via QTL analysis but was detected using the more uniform genetic backgrounds provided by NILs. Effect of *Rc* on TAC was likewise detected using NILs. Though we were unable to validate the second TAC QTL, *qPR7*, neither by MIM nor in a subset of 31 *PbPbRcRc* RILs, lack of validation does not prove nonexistence. The notably small size of the present mapping populations limited the ability to identify additional QTLs of small effect. For example, effect of *Pb* on PA was detected in NIL analysis, but was not detected through QTL mapping in 76 Rc‐RILs. Validation of *qPR7* in this second population fixed for *Rc* would be especially difficult if its effects are reduced in the presence of *Rc*. Epistasis like this was found to impact effects of *Pb* on PA, which were larger in NILs with *qPR3D* than in NILs having *qPR3L*. Lack of residual heterozygosity for *qPR7* and *qPR5* among our RILs fixed at the *Rc*, *Pb*, and *qPR3* loci prevented us from analyzing *qPR7* and *qPR5* in NILs.

In addition to QTL discovery, this study demonstrated the ability to use a non‐destructive quantitative measure of bran color purpleness to identify TAC QTLs. Previous study of 25 purple pericarp cultivars showed that the color parameter b* of the CIE L*a*b* color space was negatively correlated with TAC across purple genotypes (Table 4 in Chen et al., 2017); b* measures yellowness‐blueness with negative b* indicating blueness. In this study, we demonstrated that by specifically selecting purple pixel area, defined by the color space of hue/saturation/intensity, of the image of anthocyanin containing grains to quantify %purpleness, we obtained non‐destructive quantitative data that successfully identified *qPR3* as affecting variance in TAC among *PbPb* rices. In addition to time efficiency compared to either wet chemistry or the use of the HunterLab Miniscan XE Plus colorimeter previously used (Chen et al., 2017), which requires at least 40 g grains for the analysis, the currently used flatbed imaging analysis did not require a minimum number of grains. We did not use image analysis or visual selection for the redness of Rc‐RIL grains because previous analysis of 32 red pericarp cultivars (Chen et al., 2016) demonstrated that while the color parameter a*, which measure redness‐greenness, was positively correlated with cell‐wall associated PAs, which accounted for only 4.4% of total PAs, it was not indicative of the extractable PAs (95% of the total PA). The present study identified PA QTLs using data on extractable PAs. Furthermore, PAs can be colorless or colored, developing a red/brown color only after oxidation by flavonoid oxidase (Pourcel et al., 2005). The two *pbpbRcRc* samples in Figure 1 demonstrate differences between externally visible and extractable PA, with the grains in the upper image (*qPR3LqPR3L*) containing less PA but being visually redder than the than the *qPR3DqPR3D* grains in the image below.

Both TAC and PA mapped independently to *qPR3* between RM15123 (03_15764172 bp) and RM3400 (03_17266134 bp).Two genes in this QTL region have annotated gene functions associated with biosynthesis of anthocyanin. One is a R2R3 MYB transcription factor, *Kala3* (LOC_Os03g29614) located at chromosome 3 16,879,442 bp and previously shown to be associated with purple/black rice bran color (Maeda et al., 2014; Kim et al., 2021). The other is a *leucoanthocyanidin dioxygenase* (*LDOX*) gene (LOC_Os03g32470), located at chr3 18,570,651 bp. The predicted placement of *qPR3* between RM15123 and RM3400 suggests that the underlying gene is more likely to be *Kala3* than the nearby *LDOX*. Confirmation would require further mapping with additional recombinant progeny and biochemical analyses.

Wang et al. (2022) identified 11 *LDOX* genes in rice using BLASTP searches and deduced protein homology, two of which, LOC_Os01g27490 and LOC_Os06g42130, are also known as *OsANS1* and *OsANS2*, respectively, and have been documented to affect purple coloration and/or TAC in rice pericarps (Jung et al., 2019; Lepiniec et al., 2006; Reddy et al., 2007). Anthocyanin is synthesized from anthocyanidins, and ANS/LDOX synthesizes anthocyanidins from leucoanthocyanidins (Figure S1); thus, the LOC_Os03g32470 *ANS/LDOX* locus could be a candidate for regulation of anthocyanin. However, we also mapped PA to *qPR3*, requiring consideration of ANS/LDOX effects on PA as well as TAC. There are two branches of flavonoid biosynthetic pathway that synthesize flavan‐3‐ols, monomers of PAs, from leucoanthocyanidins; one is catalyzed by leucoanthocyanidin reductase (LAR) and the other is via ANS/LDOX to anthocyanidins and then by anthocyanidin reductase (ANR) to flavon‐3‐ols (Shih et al., 2008). The expression of *ANS/LDOX* was found upregulated in pericarp of black rice, but it was barely expressed, if at all, in red pericarp, while *LAR* was upregulated in red pericarp, not in purple pericarp (Lim & Ha, 2013; Oikawa et al., 2015). Therefore, with an inactive *ANS/LDOX*, that is, *pbpbRcRc* genotype with non‐purple red grains, PAs can be synthesized. However, in that case, PA would not be associated with *ANS/LDOX*. Our results showed that *qPR3*, tagged by RM3400, associates with PA in RILs having *pbpbRcRc* (red pericarp) (Figure S5); that is, *pbpbRcRc* RILs having *qPR3D* had significantly higher PA than those having *qPR3L*. Biochemically, *ANS/LDOX* does not fit all of our observations, making the *Kala3* MYB transcription factor a more likely candidate gene for *qPR3*.

A TAC QTL was also identified on chromosome 7, near an *anthocyanidin 3‐O‐glucosyltransferase* gene *(AGT*, LOC_Os07g32620). Kim et al. (2021) demonstrated that *OsKala3* and *OsKala4* (a.k.a., *OsB2*) mediate the activation of anthocyanin pathway synthesis genes. Further, the promoter of *Kala3* correlates strongly with the induction of its own expression and forms a positive feedback loop with its own promoter (Kim et al., 2021). A similar functional gene located on rice chromosome 6, UDP‐glucosyl transferase (UGT) (*Os06g0192100*), which along with *ANS* were specifically upregulated in black pericarp of F_1_ plants (Oikawa et al., 2015). Thus, it is possible that *qPR3* (i.e., *Kala3*) is upregulating itself in addition to increasing *qPR7* gene expression, leading to association with TAC. Though our study did not validate *qPR7*, it remains worthy of further investigation.

Like *qPR7* for TAC, the second PA QTL, *qPR5*, was not validated within this study. Use of additional recombination dissecting this QTL region to fine‐map this QTL would be required to clarify relationships between *qPR5* and the two flanking genes, *MYB* and *WRKY*, whose functions potentially impact accumulation of pigmented flavonoids.

The general model across all species analyzed to date for the regulation of flavonoid biosynthetic pathway is that the MYB transcription factor activates the EBGs while the regulation of the LBGs for anthocyanins and PAs requires the MYB‐bHLH‐WD40 (MBW) complex (Hichri et al., 2011; Koes et al., 2005; Li et al., 2014). In *Arabidopsis thaliana*, there are three MYBs activating EBGs and these are different from those activating LBGs. For the LBGs, the MYBs of *Arabidopsis* that activated anthocyanin pathway genes were different from those that activated PA biosynthesis. On the other hand, a common bHLH AtTT8 protein, a ‘B’ component of MBW, regulates both anthocyanins and PAs. Other bHLH TFs that also control both anthocyanin and PA pathways are morning glory *bHLH2* and *IVS* (Park et al., 2004, 2007). In rice, two bHLH genes (*Pb* and *Kala4*) regulate purple grain phenotype and one bHLH gene (*Rc*) controls red grain. OsKala3, also known as OsMYB3, is a MYB protein that interacts with OsKala4 to form a complex that regulates purple pericarp (Maeda et al., 2014; Zheng et al., 2021; Kim et al., 2021). Transfecting rice protoplasts with *OsKala3* and *OsKala4* together but not alone, activates anthocyanin biosynthetic genes, both EBGs and LBGs (*OsCHS*, *OsCHI*, *OsF3H*, *OsDFR*, and *OsANS1*) (Zheng et al., 2021). However, the involvement of *OsKala3* in regulating genes specifically for biosynthesis of PAs (for example, LAR) was not previously reported. Our study showed that *OsKala3* is the candidate gene underlying the *qPR3* QTL associated with PA as well as TAC. Our findings, along with previous studies on anthocyanin regulation, suggest that Kala3 is most likely the MYB component of the MBW complex for regulating pigmented flavonoid biosynthesis in rice pericarp.

Prior studies using genome‐wide association of a diverse population including white and colored‐pericarp genotypes identified QTLs for total flavonoid content on multiple chromosomes and *OsRa* (Shao et al., 2011; Xu et al., 2016). Among the previously reported QTLs for total flavonoid, only two, Os*Rc* and Os*Ra/OsPb* were co‐located with loci identified in the present study as affecting TAC or PA in Pb‐RILs or Rc‐RILs, respectively. The contents and classes of flavonoids in pigmented and non‐pigmented rices are complex, comprising multiple classes of anthocyanins, proanthocyanidins (monomers, oligomers, and polymers), flavones, flavanones, and flavonols (Chen et al., 2012; Mbanjo et al., 2020; Poulev et al., 2017).

Kim et al. (2021) reported that pericarp color was determined by the number of repeat units (RUs) in the *OsKala3* promoter region rather than differences in its coding region. Each of the RUs contained five MYB‐binding cis‐elements and five seed‐specific cis‐elements (Kim et al., 2021). In studying 40 rice cultivars (13 white, 16 black/purple, and 11 red), all black pericarp cultivars contained two RUs and all white and red pericarp cultivars contained one RU. It was concluded that the expression of *OsKala3* harboring a single RU in the promoter region was not sufficient to activate its own promoter via a positive feedback loop (Kim et al., 2021). Nonetheless, all the white grain cultivars had inactive *rc* and *kala4* and all the red grain cultivars had inactive *kala4* (Kim et al., 2021), and rice cultivars having inactive *kala4* do not accumulate anthocyanins. Therefore, red grain cultivars having inactive *kala4* and *kala3* cannot be used to prove that the one RU in the promoter of *kala3* is the cause of non‐purple grain phenotype. Zheng et al. (2021) reported similar transcript levels of *OsKala3* for red and purple pericarp, and both have higher *OsKala3* transcripts than the white pericarp. Therefore, there might be an additional mechanism involved besides the number of RUs in the promoter of *OsKala3*.

IR36ae is *pbpbrcrc* and “242” has *PbPbRcRc*. We selected Pb‐RILs and Rc‐RILs from this IR36ae x “242” mapping population using the dinucleotide functional *Pb* marker and the Rid12 marker to tag *Pb* and *Rc*, respectively. Further study of the Pb‐RILs and Rc‐RILs led to the discovery that *qPR3* regulates the TAC and PA in purple and red rice grains, and does not function as an on/off switch for these pigmented flavonoids as *Pb* and *Rc* do. However, the magnitudes of the *qPR3* allele effects on TAC and PA appeared to be different. Dividing each of these subsets by RM3400‐indicated *qPR3* alleles, the TAC of *Pb*‐RILs having *qPR3D* averaged across both years/generations was 4.7x of that RILs having *qPR3L* (Figure S4), while averaged PA of Rc‐RILs having *qPR3D* was nearly 75% higher than RILs having *qPR3L* (Figure S5). On top of that, Rc‐RILs with *qPR3L* accumulated significant PA in grains (Figure S5). *Rc* was identified as a transcription factor regulating red pericarp phenotype in two mapping populations and transgenic lines (Furukawa et al., 2007; Septiningsih et al., 2003; Sweeney et al., 2006), and no other genes or QTLs were identified in those studies, suggesting that Rc, a bHLH protein, might activate PA biosynthetic pathway genes alone. The bHLH transcription factors can individually bind to structural genes without requiring association with a MYB. For example, the petunia *Spinacia oleracea* bHLH JAF13 and *Arabidopsis* TT8 can individually bind the *SoANS* and *AtDFR* promoters alone (Shimada et al., 2006).

Both the black and red rice pericarps expressed the genes in the shared portion of the biosynthetic pathway, including the EBGs (*CHS, CHI, F3H, F3’H*) and *DFR*, a LBG (Li et al., 2014; Hichri et al., 2011; Koes et al., 2005). In this study, we assessed whether stacking *Rc* and *Pb* would cause a trade‐off between anthocyanins and proanthocyanidins due to competition for precursors. The Rc‐NILs having *qPR3D* and *PbPb* showed that NILs having *RcRc* had 2x more TAC than the *rcrc* NILs. As for the Pb‐NIL comparisons, where progeny contrasted for *Pb/pb* but were homozygous for *RcRc* and for either *qPR3D* or *qPR3L*, the PAs of active *PbPb* NILs were significantly higher than in their paired *pbpb* NILs. We demonstrated that *Rc*, a gene identified as a regulator for synthesis of PAs, elevated anthocyanin content, and conversely showed that *Pb*, a gene known as a bHLH transcription factor for anthocyanin synthesis, increased PA in pigmented rice. In addition to the specificity of *Pb* and *Rc* in regulating individual respective pathway LBGs, the Rc and Pb proteins might also be acting as a modulator by upregulating the expression of the shared genes, both EBGs and DFR, thereby accumulating more precursors and leading to the enhancement of both anthocyanins and PAs deposited in pericarps. Modulators that play a positive or negative role on the activity of MBW complex for anthocyanin synthesis have been reported (Li et al., 2014). Among them, two mechanisms that positively regulate pigmented flavonoid synthesis might explain effects of *Pb* and *Rc* on both TAC and PA. The TCP3, as a modulator, reportedly interacts with all three *Arabidopsis* R2R3‐MYBs to stimulate the transcription of EBGs, thereby increasing flavonol production (Li & Zachgo, 2013). The *Arabidopsis* TCP3 and TT1 organized the formation of MBW complexes by synergistically associating with R2R3‐MYBs, resulting in increased TAC and PA (Appelhagen et al., 2011; Li & Zachgo, 2013). In addition, bHLH proteins could directly bind DNA, that is, the G‐box of the target genes (Hichri et al., 2011).

A previous study showed that over expressing ANS/LDOX channeled the PA precursors, leucoanthocyanidins, toward the synthesis of anthocyanidins and resulted in the increase of anthocyanins and the decrease of PAs (Reddy et al., 2007). In contrast, our data show a mutual enhancement of TAC and PA from *Pb* and *Rc* rather than a trade‐off. If these genes are modulating the expression of EBGs, then the supply of precursors for the biosynthesis of PAs and TAC would not be limited, and thus would enhance both TAC and PA rather than one at the expense of the other (Oikawa et al., 2015). Our findings indicate that rice cultivars with increased content of health beneficial flavonoids can be developed by combining both *Pb* and *Rc* along with three antioxidant‐enhancing QTLs (*qPR3, qPR5, qPR7*) for which genetic markers to facilitate marker‐assisted breeding are now provided.

## 5 CONCLUSION

Genetic mapping of RILs from IR36ae x “242” revealed three QTLs (*qPR3, qPR5, qPR7*) that regulate the concentrations of anthocyanins and proanthocyanidins, the purple and red pigmented flavonoids in pericarp of rice grains whose synthesis is turned on by the *Pb* and *Rc* transcription factors, respectively. The candidate gene for the QTL with largest effect, *qPR3*, is a MYB transcription factor for purple/black grains gene variably called *OsMYB3*. We demonstrated via QTL mapping and again in NILs that the *qPR3* allele from “242,” the parent with colored bran increased the concentrations of anthocyanins that were deposited in the grain pericarp, and was dominant, while the allele for reduced pigmentation from IR36ae was recessive. Our study further showed that this candidate MYB transcription factor at *qPR3* regulated the concentrations of proanthocyanidins in rice grains containing active *Rc*, a bHLH transcription factor gene previously known for turning on and off the proanthocyanidin biosynthesis. Additionally, the *Rc* and *Pb* genes, which activate the synthesis of proanthocyanidins and anthocyanins, respectively, were found to positively modulate the depositions of both anthocyanins and proanthocyanidins. The mutual enhancement of both anthocyanin and proanthocyanidin concentrations by both *Pb* and *Rc* further demonstrates that rice breeders can maximize antioxidants in rice grains by combining these genes, without concern about limited precursor production causing a trade‐off between anthocyanin and anthocyanidin contents.

## AUTHOR CONTRIBUTIONS

**Ming‐Hsuan Chen**: conceptualization; supervision; investigation; methodology; resources; data curation; formal analysis; and writing–original draft. **Shannon R. M. Pinson**: conceptualization; supervision; investigation; methodology; resources; and writing–original draft. **Aaron K. Jackson**: formal analysis and writing–review. **Jeremy D. Edwards**: methodology; formal analysis; and writing–review.

## CONFLICT OF INTEREST STATEMENT

The authors declare that the research was conducted in the absence of any commercial or financial relationships that could be construed as a potential conflict of interest. Mention of a trademark or proprietary product does not constitute a guarantee or warranty of the product by the USDA or Louisiana State University and does not imply its approval to the exclusion of other products that also can be suitable. The USDA is an equal opportunity provider and employer. All experiments complied with the current laws of the United States, the country in which they were performed.

## Supporting information


**Supplemental Figure S1**. The anthocyanin and proanthocyanidin biosynthetic pathways. Red font indicates the early biosynthesis genes (EBGs), blue font indicates late biosynthesis genes (LBGs) (Li, 2014), and the underlined are common genes shared by anthocyanin and proanthocyanidin biosynthetic pathways. CHS, chalcone synthase; CHI, chalcone isomerase; F3H, flavonoid 3‐hydroxylase; F3’H, flavonoid 3′‐hydroxylase; DFR, dihydroflavonol reductase; ANS, anthocyanidin synthase; LAR, leucoanthocyanidin reductase; ANR, anthocyanidin reductase; UGT, UDP‐glucosyl transferase.
**Supplemental Figure S2**. Results from composite interval mapping (CIM LS, a, b, d) and multiple interval mapping (MIM, c) of data for %purpleness defined as percentage of imaged grain area having dark (purple) color, and for total anthocyanin content (TAC, μg g^−1^) measured in grains harvested in 2019 (a—c) or averaged across 2019 and 2020 (d). Upper graphs are LOD scores lower graphs show additive effects, with negative additive effects indicating that the “242” allele increased the trait value. The 2019 data analyses (a—c) used a genetic map with 259 non‐redundant SNP loci genotyped using the 1k‐RiCA V1 panel (https://doi.org/10.1186/s12284‐019‐0311‐0), and QTLs were declared based on CIM LOD thresholds of 3.5, 4.0, and 4.5 for the α = 0.10, 0.05, and 0.01 levels, respectively. The TAC 2‐year BLUPs (d) were analyzed using a denser genetic map (401 non‐redundant SNPs) genotyped using the 1k‐RiCA V4 (https://www.agriplexgenomics.com/irri‐v4) (Arbelaez et al., 2019) and the LSU500 (Cerioli et al., 2022) panels, and had LOD thresholds of 4.0, 4.4, and 5.2 at the α = 0.10, 0.05, and 0.01 levels, respectively. Dashed lines indicate the α = 0.10 LOD thresholds for each study. CIM LS and MIM were analyzed using QGene 4.4.0.
**Supplemental Figure S3**. Results from composite interval mappings (CIM LS) using data for total proanthocyanidin content (PA, μg g^−1^) measured in grains harvested in 2019 and 2020, analyzed using QGene 4.4.0. Upper graphs are LOD scores, lower graphs show additive effects, with negative additive effects indicating that the “242” allele increased the trait value. The genetic map contained 401 non‐redundant SNPs genotyped with the 1k‐RiCA V4 (https://www.agriplexgenomics.com/irri‐v4) (Arbelaez et al., 2019) and the LSU500 (Cerioli et al., 2022) panels. QTLs were declared based on LOD thresholds of 3.5, 3.8, and 4.2 for the α = 0.10, 0.05, and 0.01 levels, respectively. Dashed lines indicate the α = 0.10 LOD thresholds.
**Supplemental Figure S4**. Means comparison of total anthocyanin content (TAC, μg g^−1^ grain) among the four genetic variants combining *Rc/rc* and *qPR3D/qPR3L* alleles (all are *PbPb*). (a) and (b) are F5 and F6 Pb‐RILs, respectively. The four genotypic classes are indicated. Different letters for the means indicate significant difference among the genetic variants at 95% confidence level using Fisher LSD method. The number of RILs in each of the genetic variant classes are as follows: *rcrc‐qPR3DqPR3D*, n = 18; *rcrc‐qPR3LqPR3L*, n = 51; *RcRc‐qPR3DqPR3D*, n = 28; and *RcRc‐qPR3LqPR3L*, n = 46.
**Supplemental Figure S5**. Means comparison of total proanthocyanidin content (PA, μg g^−1^ grain) among the four genetic variants combining *Pb/pb* and *qPR3D/qPR3L* alleles (all are *RcRc)*. (a) and (b) are F5 and F6 Rc‐RILs, respectively. The four genotypic classes are indicated. Different letters for the means indicate significantly different between the genetic variants at 95% confidence level using Fisher LSD method. The number of RILs in each of the genetic variant classes are as follows: *PbPb‐qPR3DqPR3D*, n = 30; *PbPb‐qPR3LqPR3L*, n = 43; *pbpb‐qPR3DqPR3D*, n = 41; and *pbpb‐qPR3LqPR3L*, n = 57.
