# Supplementary material for: Genetic loci regulating the concentrations of anthocyanins and proanthocyanidins in the pericarps of purple and red rice
Source: Plant Genome. 2023 May 13;16(2):e20338. doi: 10.1002/tpg2.20338 (PMC12807128; doi:10.1002/tpg2.20338)
Supplement: Supplementary file 1 [file TPG2-16-e20338-s001.pdf]

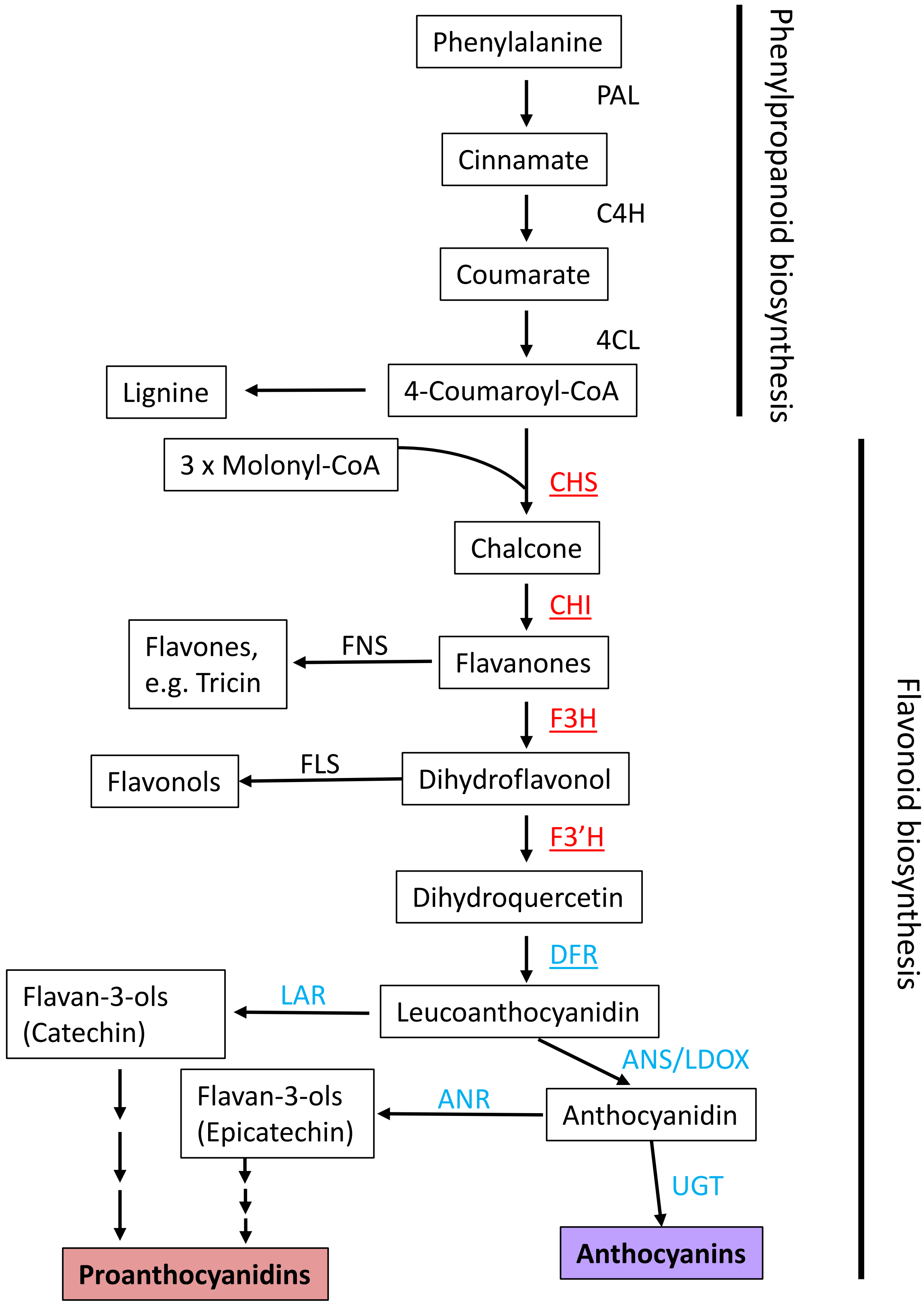

Supplemental Figure S1. The anthocyanin and proanthocyanidin biosynthetic pathways. Red font indicates the early biosynthesis genes (EBGs), blue font indicates late biosynthesis genes (LBGs) (Li, 2014), and the underlined are common genes shared by anthocyanin and proanthocyanidin biosynthetic pathways. CHS, chalcone synthase; CHI, chalcone isomerase; F3H, flavonoid 3-hydroxylase; F3'H, flavonoid 3'-hydroxylase; DFR, dihydroflavonol reductase; ANS, anthocyanidin synthase; LAR, leucoanthocyanidin reductase; ANR, anthocyanidin reductase; UGT, UDP-glucosyl transferase.

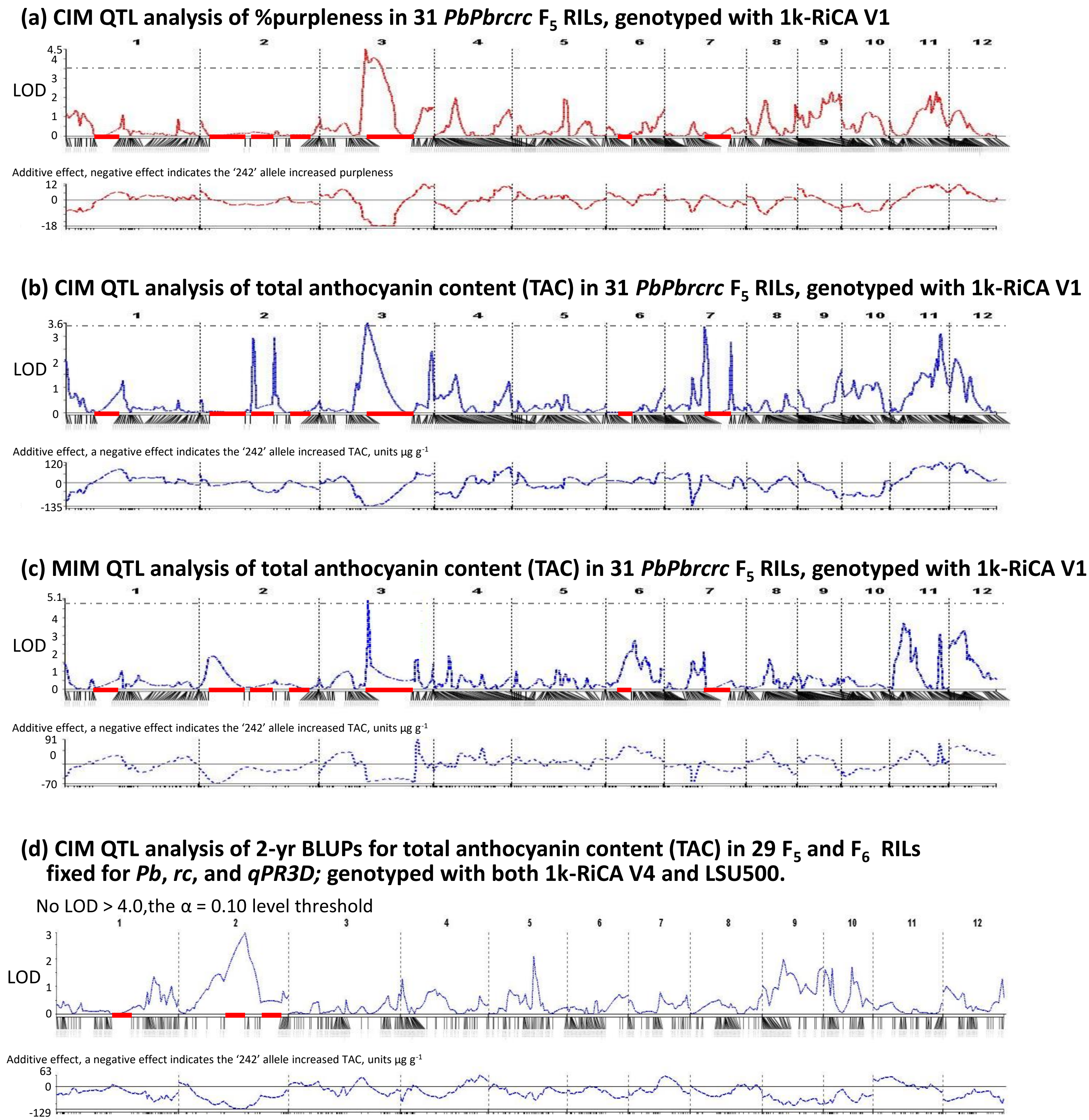

Supplemental Figure S2. Results from composite interval mapping (CIM LS, a, b, d) and multiple interval mapping (MIM, c) of data for %purpleness defined as percentage of imaged grain area having dark (purple) color, and for total anthocyanin content (TAC, μg g^{-1}) measured in grains harvested in 2019 (a - c) or averaged across 2019 and 2020 (d). Upper graphs are LOD scores lower graphs show additive effects, with negative additive effects indicating that the '242' allele increased the trait value. The 2019 data analyses (a - c) used a genetic map with 259 non-redundant SNP loci genotyped using the 1k-RiCA V1 panel (https://doi.org/10.1186/s12284-019-0311-0), and QTLs were declared based on CIM LOD thresholds of 3.5, 4.0, and 4.5 for the α = 0.10, 0.05, and 0.01 levels, respectively. The TAC 2-year BLUPs (d) were analyzed using a denser genetic map (401 non-redundant SNPs) genotyped using the 1k-RiCA V4 (https://www.agriplexgenomics.com/irri-v4) (Arbelaez et al., 2019) and the LSU500 (Cerioli et al., 2022) panels, and had LOD thresholds of 4.0, 4.4, and 5.2 at the α = 0.10, 0.05, and 0.01 levels, respectively. Marker gaps greater than 20.0 cM are indicated with red bars. Dashed lines indicate the α = 0.10 LOD thresholds for each study. CIM LS and MIM were analyzed using QGene 4.4.0.

(a) QTL analysis of proanthocyanidin (PA) measured in 75 *RcRc* F_5 RILs harvested 2019

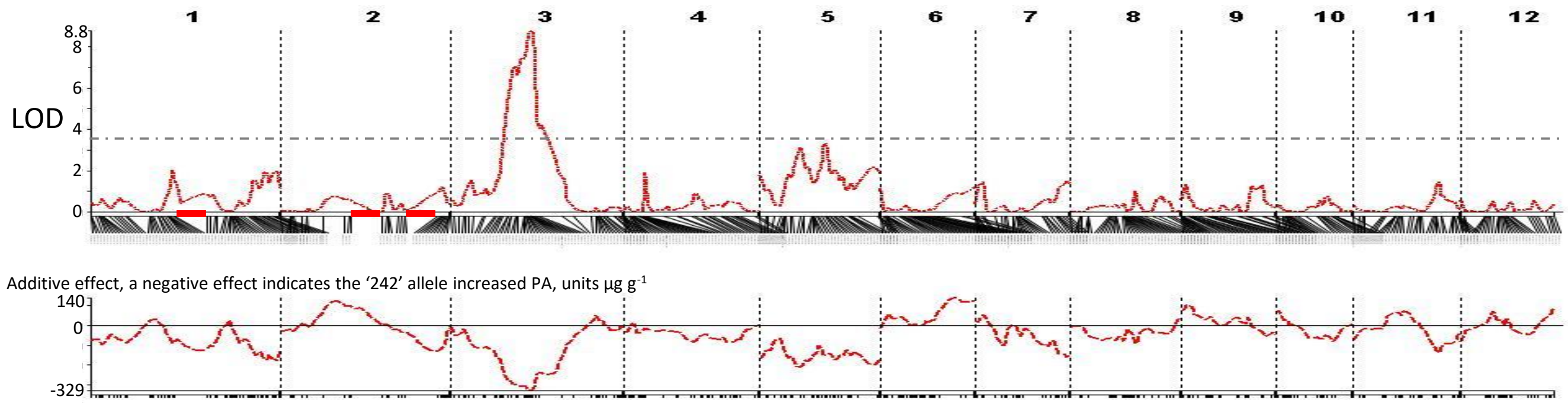

(b) QTL analysis of proanthocyanidin (PA) measured in 75 *RcRc* F_6 RILs harvested 2020

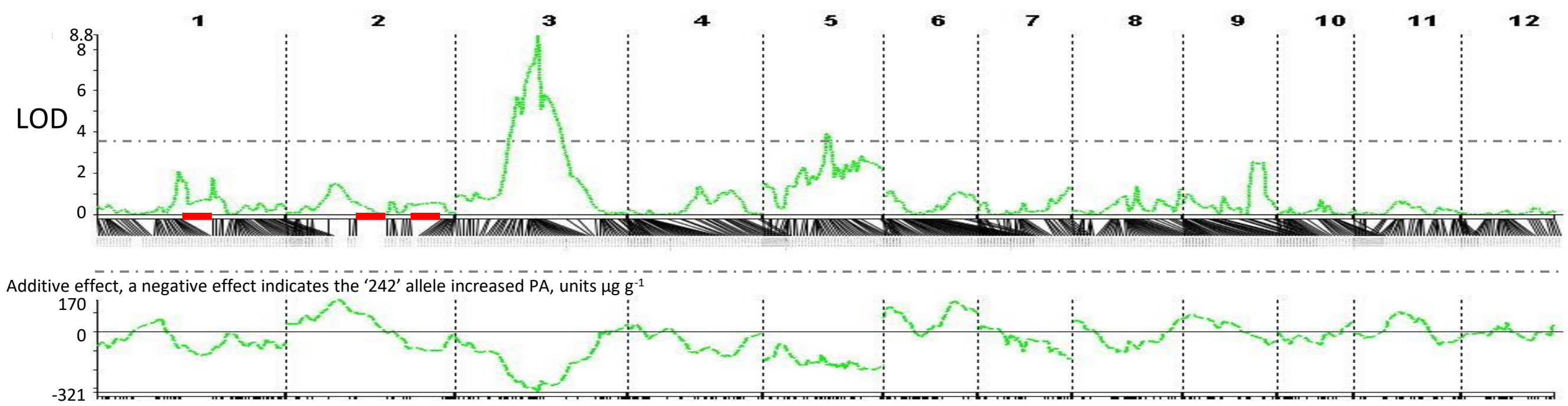

(c) QTL analysis of proanthocyanidin (PA) 2-yr trait BLUPs calculated across 2019 and 2020

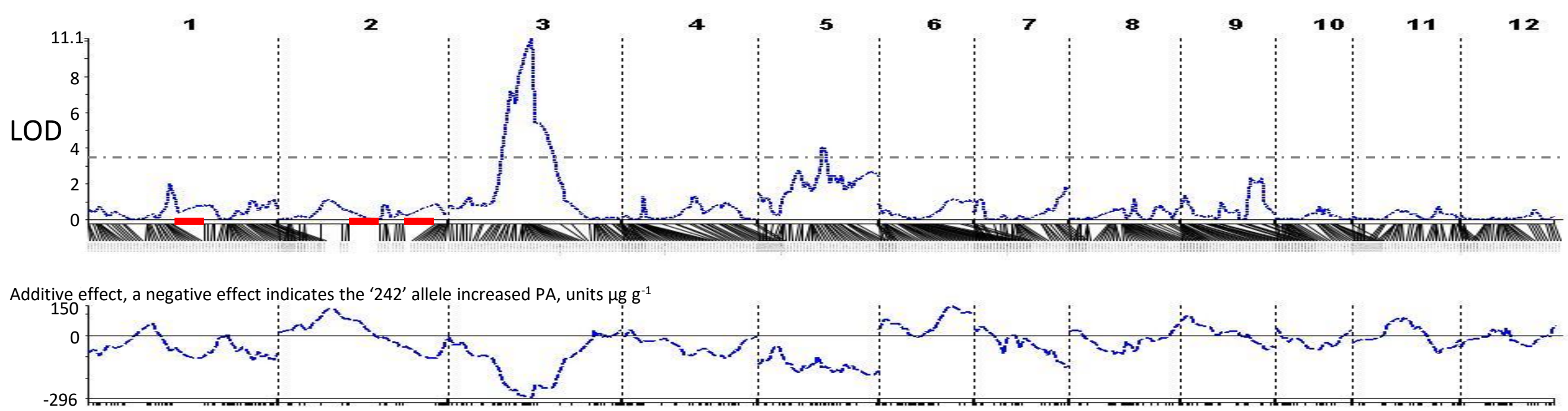

Supplemental Figure S3. Results from composite interval mappings (CIM LS) using data for total proanthocyanidin content (PA, µg g^{-1}) measured in grains harvested in 2019 and 2020, analyzed using QGene 4.4.0. Upper graphs are LOD scores, lower graphs show additive effects, with negative additive effects indicating that the '242' allele increased the trait value. The genetic map contained 401 non-redundant SNPs genotyped with the 1k-RiCA V4 (https://www.agriplexgenomics.com/irri-v4) (Arbelaez et al., 2019) and the LSU500 (Cerioli et al., 2022) panels. Marker gaps greater than 20.0 cM are indicated with red bars. QTLs were declared based on LOD thresholds of 3.5, 3.8, and 4.2 for the α = 0.10, 0.05, and 0.01 levels, respectively. Dashed lines indicate the α = 0.10 LOD thresholds.

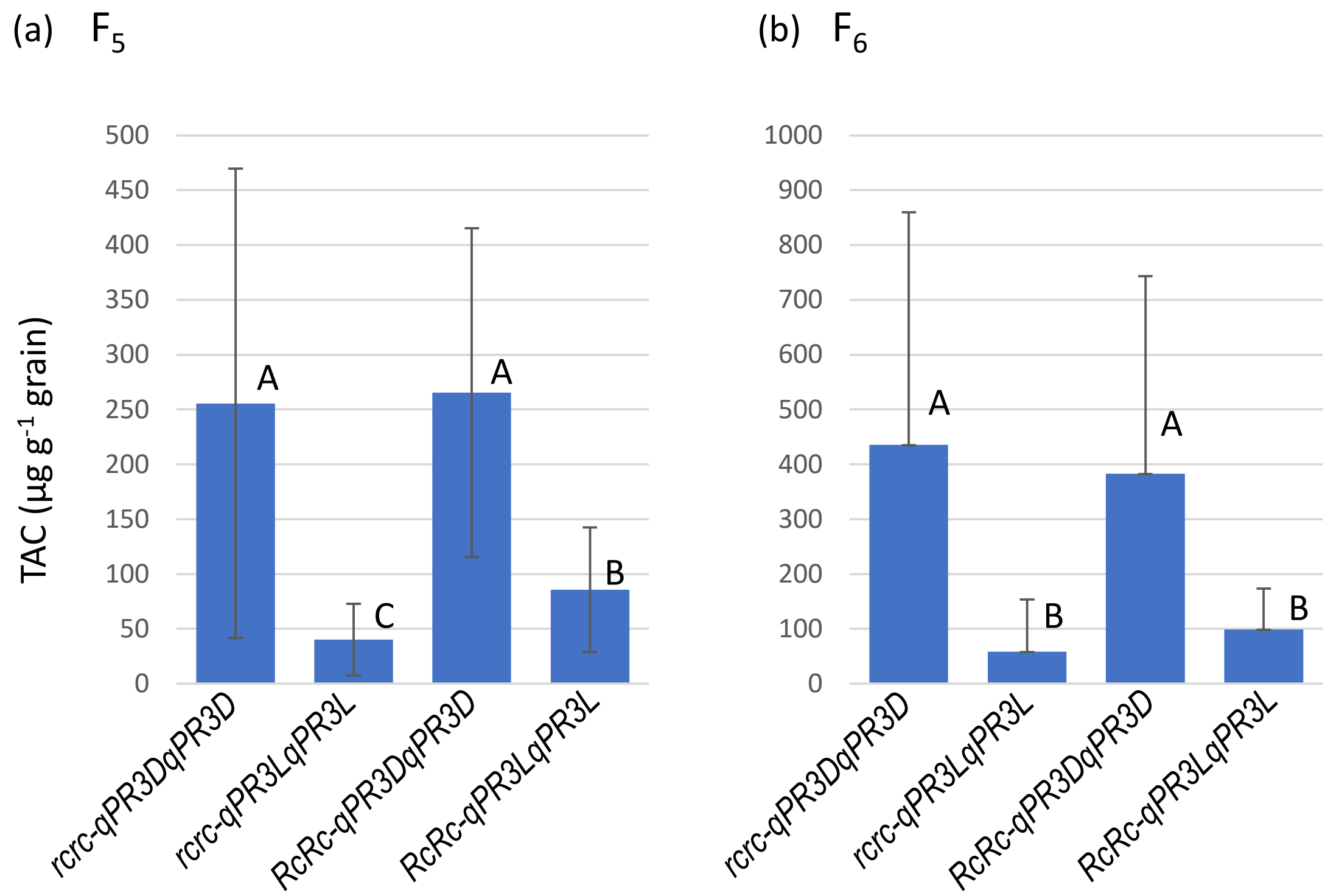

Supplemental Figure S4. Means comparison of total anthocyanin content (TAC, µg g^{-1} grain) among the four genetic variants combining *Rc/rc* and *qPR3D/qPR3L* alleles (all are *PbPb*). (a) and (b) are F_5 and F_6 Pb-RILs observed in 2019 and 2020, respectively. The four genotypic classes are indicated. Different letters for the means indicate significant difference among the genetic variants at 95% confidence level using Fisher LSD method. The number of RILs in each of the genetic variant classes are as follows: *rcrc-qPR3DqPR3D*, n=18; *rcrc-qPR3LqPR3L*, n=51; *RcRc-qPR3DqPR3D*, n=28; and *RcRc-qPR3LqPR3L*, n=46.

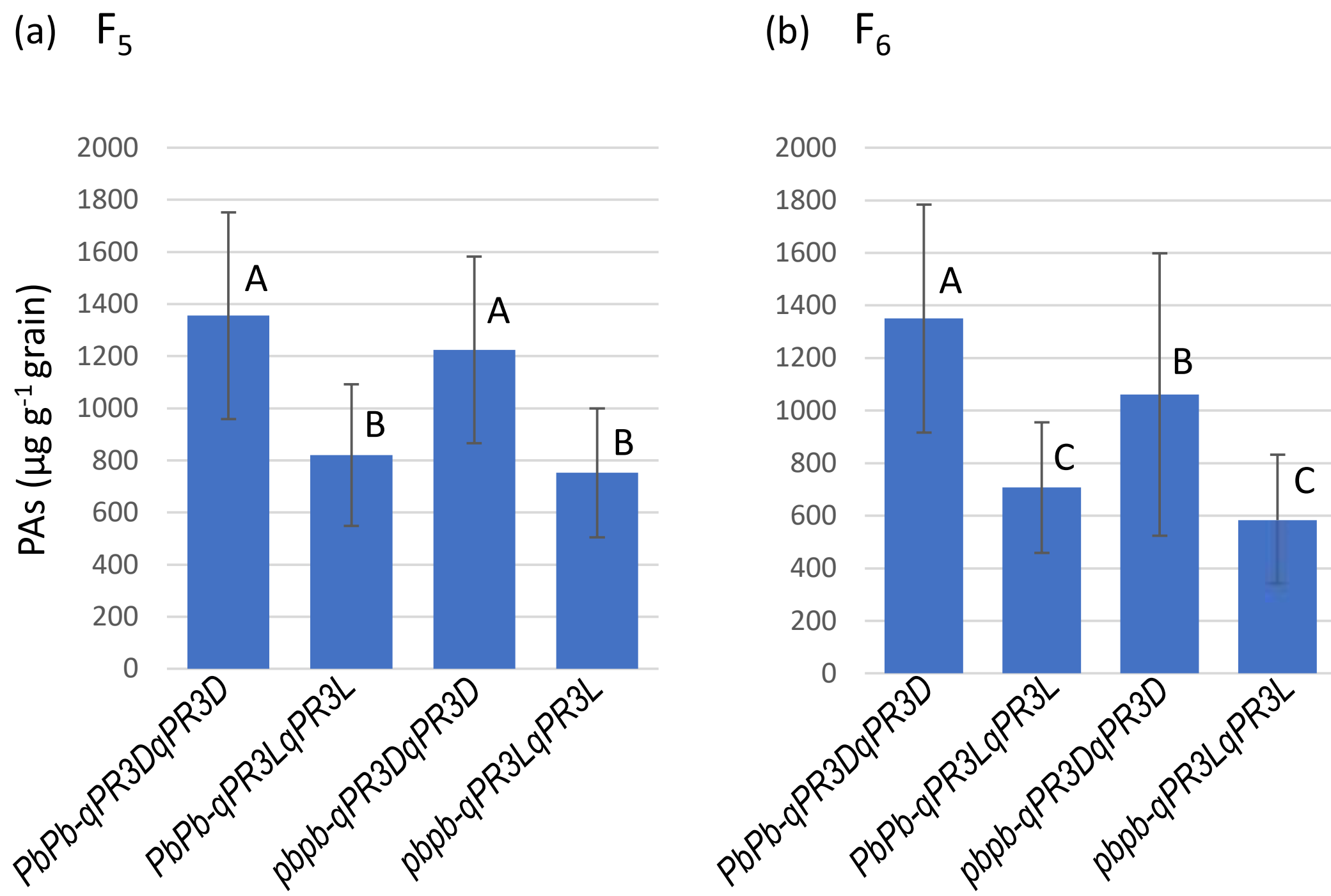

Supplemental Figure S5. Means comparison of total proanthocyanidin content (PA, µg g^{-1} grain) among the four genetic variants combining *Pb/pb* and *qPR3D/qPR3L* alleles (all are *RcRc)*. (a) and (b) are F_5 and F_6 Rc-RILs observed in 2019 and 2020, respectively. The four genotypic classes are indicated. Different letters for the means indicate significantly different between the genetic variants at 95% confidence level using Fisher LSD method. The number of RILs in each of the genetic variant classes are as follows: *PbPb-qPR3DqPR3D*, n=30; *PbPb-qPR3LqPR3L*, n=43; *pbpb-qPR3DqPR3D*, n=41; and *pbpb-qPR3LqPR3L*, n=57.
